# Cyst stem cell lineage eIF5 non-autonomously prevents testicular germ cell tumor formation via eIF1A/eIF2γ-mediated pre-initiation complex

**DOI:** 10.1186/s13287-022-03025-5

**Published:** 2022-07-26

**Authors:** Zhiran Li, Yunhao Wu, Yangbo Fu, Xia Chen, Xi Zhao, Xiaolong Wu, Yajuan Lu, Hui He, Cong Shen, Bo Zheng, Jun Yu, Fei Sun

**Affiliations:** 1 Institute of Reproductive Medicine, Medical School, Nantong University, Nantong, 226001 China; 2 Department of Obstetrics and Gynecology, Affiliated Hospital 2 of Nantong University and First People’s Hospital of Nantong City, Nantong, 226001 China; 3 Department of Human Anatomy, Medical School, Nantong University, Nantong, 226001 China; 4 State Key Laboratory of Reproductive Medicine, Center for Reproduction and Genetics, Suzhou Municipal Hospital, The Affiliated Suzhou Hospital of Nanjing Medical University, Gusu School, Nanjing Medical University, Suzhou, 215002 China

**Keywords:** Translation initiation, eIF5, Testicular germ cell tumor, Stem cell niche, Differentiation

## Abstract

**Background:**

Stem cell niche maintains stem cell population identity and is essential for the homeostasis of self-renewal and differentiation in *Drosophila* testes. However, the mechanisms of CySC lineage signals-mediated soma–germline communications in response to external stimuli are unclear.

**Methods:**

Pre-initiation complex functions were evaluated by UAS-Gal4-mediated cell effects. RNA sequencing was conducted in NC and *eIF5* siRNA-treated cells. Genetic interaction analysis was used to indicate the relationships between eIF5 and eIF1A/eIF2γ in *Drosophila* testes.

**Results:**

Here, we demonstrated that in CySCs, translation initiation factor eIF5 mediates cyst cell differentiation and the non-autonomously affected germ cell differentiation process. CySCs lacking eIF5 displayed unbalanced cell proliferation and apoptosis, forming testicular germ cell tumors (TGCTs) during spermatogenesis. eIF5 transcriptional regulation network analysis identified multiple metabolic processes and several key factors that might be involved in germ cell differentiation and TGCT formation. Importantly, knockdown of eIF1A and eIF2γ, key components of pre-initiation complex, mimicked the phenotype of knocking down eIF5 in the stem cell niche of *Drosophila* testes. Genetic interaction analysis indicated that eIF5 was sufficient to rescue the phenotype of tumorlike structures induced by down-regulating eIF1A or eIF2γ in CySCs.

**Conclusions:**

These findings demonstrated that CySC lineage eIF5, together with eIF1A or eIF2γ, mediates soma–germline communications for the stem cell niche homeostasis in *Drosophila* testes, providing new insights for the prevention of TGCTs.

**Supplementary Information:**

The online version contains supplementary material available at 10.1186/s13287-022-03025-5.

## Introduction

Testicular germ cell tumors (TGCTs) are the most common form of solid cancer in young males, which are related to the formation of the germ cell pool and gametogenesis [1, 2]. The balance between self-renewal and differentiation of germline stem cells (GSCs) in the adult testis is essential to maintain normal spermatogenesis and fertility throughout life [2]. TGCTs are thought to originate from a germ cell lineage that is blocked at differentiation and maturation and are highly conserved in mammalian and *Drosophila* testes [3, 4]. Previously, scholars found that Sertoli cells ectopically expressing glial cell line-derived neurotrophic factor (GDNF), a spermatogonial stem cell (SSC) factor for attracting SSCs to niches in mice testes, could induce proliferated clusters of undifferentiated spermatogonia, which finally resulted in the formation of non-metastatic tumors [5–7]. Similarly, TGCTs can also be formed in *Drosophila* when mitotic germ cells fail to differentiate and over-proliferate [4]. However, the causative genetic aberrations and pathogenic mechanisms are poorly understood.

In *Drosophila* testes, soma–germline communications are tightly controlled by the stem cell niche, which plays key roles in the homeostasis of self-renewal and differentiation [8, 9]. The cyst stem cell (CySC) lineage coordinates with GSCs to balance the self-renewal capacity with differentiation [9]. The homeostasis and coordination of soma–germline communications are achieved by the stem cell niche together with integrated signals [10]. GSC differentiation is repressed by bone morphogenetic protein (BMP) signaling, which suppresses *bag of marbles* (*bam*) expression within the stem cell niche [11, 12]. Importantly, an aberrant reduction in the Bam level can generate extra GSC-like masses in *Drosophila* testes [13]. Previously, Yu and his colleagues performed a large-scale screen in *Drosophila* testes and identified 221 regulators of GSC maintenance or differentiation [14]. Among these regulators, we identified eukaryotic translation initiation factor eIF5, which mediates protein synthesis, as a potential regulator of the stem cell niche that exhibits significant roles in soma–germline communications in *Drosophila* testes.

In eukaryotes, mRNA translation is a complicated process that includes four major phases (initiation, elongation, termination, and ribosome recycling) [15]. As described previously, mRNA translation is primarily regulated at translation initiation phase, which begins with the formation of the eIF2-GTP-Met-tRNAi^Met^ ternary complex, which then recruits translation initiation factors (eIFs) such as eIF1, eIF1A, eIF3, eIF5, and the 40S ribosomal subunit, to form the 43S pre-initiation complex [16]. After the recognition of the initiation codon and formation of the 48S initiation complex, eIF5, which is a GTPase activating protein (GAP), promotes the hydrolysis of the eIF2-GTP complex, leading to the displacement of eIFs and joining of a 60S ribosomal subunit [17]. Dysfunction of eIFs (e.g., eIF4, eIF3, and eIF2) are involved in birth defects, infertility, and various types of cancer [16, 18–21]. Moreover, mRNA translation is of great significance for GSCs to guide their differentiation into sperm, and eIFs play key roles in the regulation of protein synthesis, ultimately completing such cell fate decisions [21].

In this study, we aimed to investigate the roles of eIF5 in the regulation of soma–germline communications in the stem cell niche of *Drosophila* testes. We provided evidence that eIF5 in CySC lineage, together with eIF1A and eIF2γ, promotes cyst cell and germ cell differentiation, which ultimately prevent the formation of TGCTs.

## Materials and methods

### Fly stocks and fly crosses

All fly strains were cultured on standard cornmeal food at 25 °C and grown in a proper humidity. The transgenic RNA interference (RNAi) and tool flies were obtained from TsingHua Fly Center (THFC) as follows: UAS-eIF5 RNAi (THU0690), UAS-eIF1A RNAi (THU4000), UAS-eIF2γ RNAi (THU1956), and Ptc-Gal4 UAS-GFP; tub-Gal80^ts^ (THJ0205). Tj-Gal4 (#104055) was acquired from *Drosophila* Genetic Resource Consortium (DGRC). The W^1118^ line was used as control. The construction of transgenic fly strain (UAS-eIF5 OE) was assisted by Core Facility of *Drosophila* Resource and Technology, CEMCS, CAS.

Two- to three-day-old flies were selected for mating in this study. The UAS/Gal4 crosses were set and raised at 25 °C. Male Tj-Gal4 drivers were crossed to the transgenic virgin females. And then qualified male offspring with specific genotypes were chosen for the further functional analysis. Temperature-sensitive Ptc-Gal4 male flies were crossed with UAS-RNAi virgin females at 25 °C. After egg laying, cultures were transferred to 18 °C until adults emerged. Newly enclosed males were shifted to high temperature (29 °C) for 0, 9 and 15 days.

### Plasmid construction

eIF5 CDS was subcloned into the pUAS-attB-3xHA vector, and the CDS sequence was amplified by PCR using primers to introduce NotI (1166A, Takara, Shiga, Japan) and XbaI (1093A, Takara, Shiga, Japan) restriction sites. Plasmid construction protocol by classical restriction ligation cloning has been described before [22]. The following primers were used: F: 5-ATAAGAATGCGGCCGCTATGGCCACCGTAAACGTAAACC-3, R: 5-GCTCTAGATTAGATATCGTCGATGTTCAC-3.

### Cell culture and transfection

*Drosophila* Schneider 2 (S2) cells were cultured in Schneider’s *Drosophila* medium (21720024, Gibco, USA) supplemented with 10% heat-inactivated fetal bovine serum (FBS) (04-001-1ACS, Bioind, Israel) at 28 °C. S2 cells were split with supplemented medium at a ratio of 1:3 every 3–4 days.

S2 cells were seeded into a six-well plate, and transfection was carried out when the cell growth area reached 70–80% of the well. Lipofectamine 2000 Transfection Reagent (Lipo2000, 11668019, Invitrogen, Waltham, MA, USA), Opti-Minimal Essential Medium (MEM) (31985-062, Gibco, USA), and siRNA were used together to knock down target genes. Transfection was performed according to the following process: Two tubes were prepared to mix reagent, one contained 250 μL Opti-MEM and 15 μL Lipo2000 and the other contained 250 μL Opti-MEM and 15 μL siRNA, and incubated for 5 min after vortexing for 5 s at room temperature, and then mixed with two tubes and incubated for 20 min after vortexing for 5 s at room temperature. The GenePharma Company (Suzhou, China) was responsible for the design and synthesis of the siRNAs. The detailed information of siRNA is listed in Additional file 2: Table S1.

### Quantitative reverse transcription PCR (qRT-PCR)

Total RNA was extracted using TRIzol Reagent (15596026, Invitrogen, Waltham, MA, USA) from testes and S2 cells according to the manufacturer’s protocol. Using PrimeScript™ II 1st Strand cDNA Synthesis Kit (6210A, Takara, Shiga, Japan) to synthesize cDNA. qRT-PCR was performed in LightCycler® 96 Real-Time PCR System (Roche) using TB Green Premix Ex Taq II (RR820, Takara, Shiga, Japan). The detailed primer sequences are shown in Additional file 3: Table S2.

### Immunofluorescence

Fly testes were dissected in 1 × phosphate-buffered saline (PBS) and fixed for 30 min in 4% paraformaldehyde (PFA). They were washed three times with 0.3% PBS-Triton X-100 (PBST) and incubated in 5% bovine serum albumin (BSA) for 30 min. Primary antibodies (Additional file 4: Table S3) were diluted in 5% BSA solution, and testes were incubated at room temperature for 1 h and then washed three times in 0.3% PBST. Secondary antibodies were conjugated with A488, Cy3, or A647 (Jackson ImmunoResearch Laboratories, West Grove, PA, USA) and were diluted at a ratio of 1:400 with 5% BSA, and incubated at room temperature for 1 h avoiding light. Testes were then washed three times again by 0.3% PBST and stained with Hoechst 33342 (1.0 mg/mL, C0031, Solarbio, Beijing, China) for 5 min before finalizing. According to the manufacturer’s instructions, F-actin and EdU staining were performed with Alexa Fluor™ Plus 555 Phalloidin (1:50; A30106, Invitrogen, Waltham, MA, USA) and Cell-Light™ EdU Apollo488 In Vitro Kit (C10310-3, RiboBio, Guangzhou, China), respectively.

### TUNEL assay

Cell apoptosis was performed by One-Step TUNEL Apoptosis Assay Kit (C1090, Beyotime, Shanghai, China) according to the manufacturer’s protocols. Fly testes or S2 cells were fixed for 20 min in 4% PFA and washed with 0.5% PBST for three times. The mixture of 45 μL fluorescent labeling solution and 5 μL TdT enzyme was prepared in the dark. Fly testes or S2 cells were incubated with the mixture for 1 h at 37 °C in the dark and washed in 1 × PBS three times before staining with Hoechst 33342 (1.0 mg/mL, C0031, Solarbio, Beijing, China).

### Cell viability assay

CCK-8 Cell Counting Kit (A311-01-AA, Vazyme, Nanjing, China) was utilized to assess cell growth situation according to the manufacturer’s protocols. Briefly, transfected S2 cells were transferred to 96-well plates (3000 cells per well) and incubated in 10% CCK-8 reagent that was diluted in Schneider’s *Drosophila* medium at 37 °C for 2 h. After transfection at 0, 24, 48, and 72 h, the absorbance in each well was evaluated at 450 nm (FlexStation® 3, Molecular Devices, California, USA). All experiments were repeated at least three times.

### RNA isolation and RNA sequencing

Total RNA was extracted from S2 cells using TRIzol (15596026, Invitrogen, Waltham, MA, USA). The RNA concentration and purity were detected by the NanoDrop instrument (Thermo Fisher Scientific, Waltham, Massachusetts, USA), and the integrity of the RNA was detected by gel electrophoresis, and the RNA integrity (RIN) values were measured using an Agilent 2100 instrument (Agilent Technologies, Santa Clara, CA, USA). Libraries for indexed RNA-Seq were prepared from 800 ng of total RNA using the TruSeq RNA Library Prep Kit v2 (Illumina, San Diego, CA, USA) following the manufacturer’s protocol. The following steps of purification of poly (A) mRNA with oligo-dT magnetic beads, RNA fragmentation, synthesis of double-stranded cDNA using SuperScript II Reverse Transcriptase (Invitrogen, Waltham, MA, USA), ligation of indexed Illumina adapters, and amplification using limited-cycle PCR were included in this experiment. Sequencing libraries were validated by capillary electrophoresis using a Bioanalyzer 2100 instrument (Agilent Technologies). The DNB (DNA nanoball) was prepared after the libraries were tested for qualification, and then loaded into the sequencing chip. Sequencing was performed using a high-throughput sequencer (MGIseq2000, MGI, Shenzhen, China).

### Bioinformatic analysis for RNA-seq

Reads were aligned to the *Drosophila* melanogaster reference genome in NCBI’s assembly resource (www.ncbi.nlm.nih.gov/assembly/) using the Bowtie package with Hierarchical Indexing for Spliced Alignment of Transcripts (HISAT) comparison software [23]. The transcript abundances were determined using Fragments Per Kilobase per Million mapped reads (FPKM) values with the RSEM tool [24]. Among all the identified genes, those with extremely low expression values (mean values below 0.1) were filtered out. Differentially expressed genes were identified using criteria of FDR < 0.05 and |FC|> 1.5. Expression-related graphs were plotted by using the R software (version: 4.0.2). The clustered heatmap, Circos plot, and volcano plot were implemented by the pheatmap, circlize, and ggplot2 package, correspondingly. The STRING database (version 11.0) was applied for gene functional enrichment and gene-to-gene interaction analyses [25]. Functional annotations from the GO and KEGG pathway databases were used for enrichment analysis, and FDR < 0.05 was considered statistically significant. The Cytoscape software (version 3.8.2) was further used to refine and construct complex network of gene-to-gene interaction, gene-to-function, and gene expression changes [26]. GSEA was performed using the GSEA function embedded in the cluster Profiler package (version 3.18.0) in the R software [27]. *P* < 0.05, FDR < 0.25, |NES| > 1 were set as the cutoff for a statistically significant enrichment.

### Statistical analysis for functional experiments

All the functional experiments conducted in this study were repeated at least three times. The quantitative results were evaluated for statistical differences using Student’s t test by GraphPad Prism software Version 6.01 (GraphPad Inc., La Jolla, CA, USA). * *P* < 0.05; ** *P* < 0.01; *** *P* < 0.001.

## Results

### CySC lineage eIF5 mediates cyst cell and germ cell differentiation

**Fig. 1:**
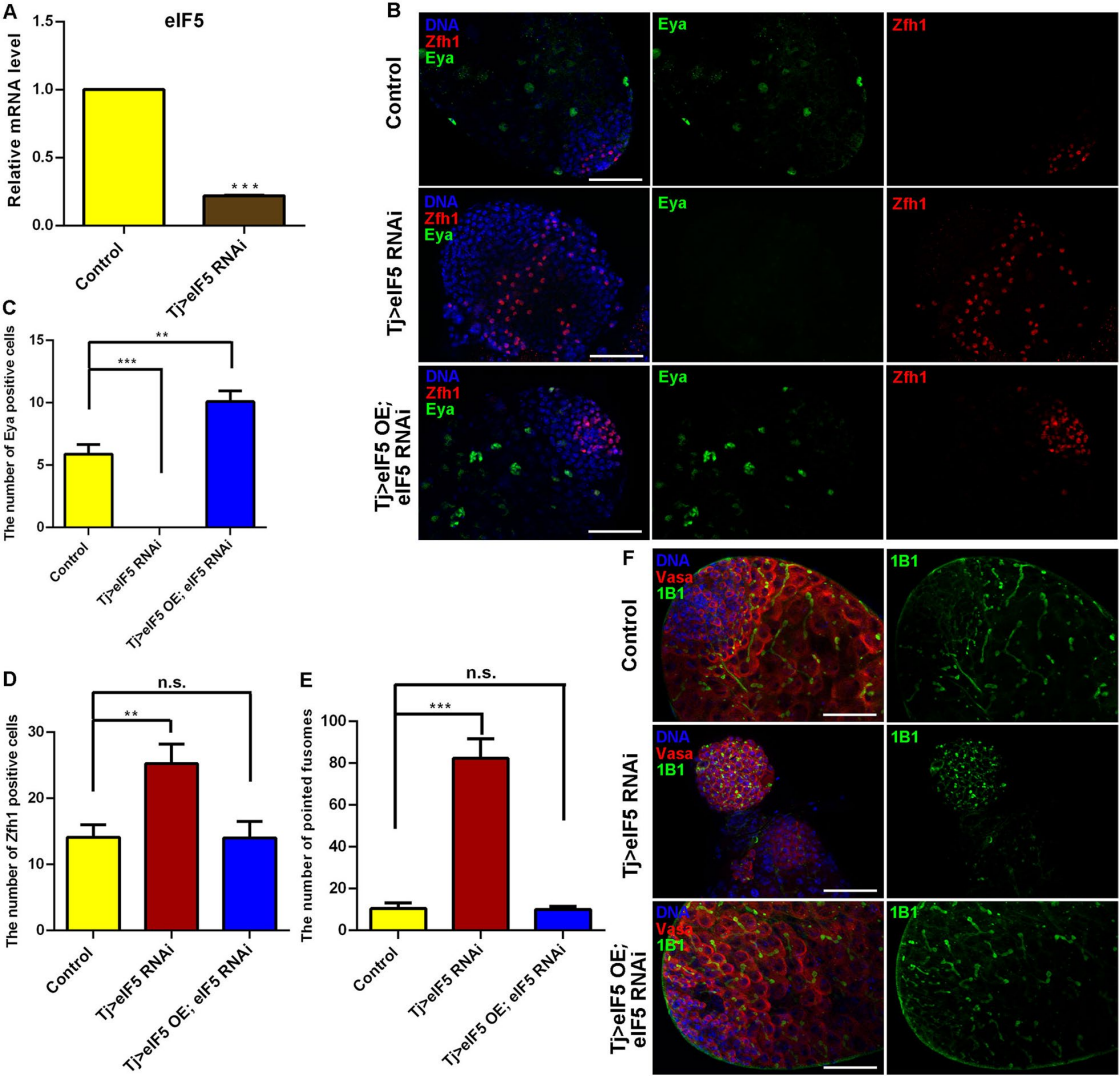
Reduction in eIF5 in CySCs blocked cyst cell differentiation and caused GSC-like mass accumulation. **A** Relative mRNA level of *eIF5* in control and Tj > eIF5 RNAi testes. **B** Immunostaining of Zfh1 (red) and Eya (green) at the apex of control, Tj > eIF5 RNAi, and Tj > eIF5 OE; eIF5 RNAi testes. **C** The number of Eya-positive cells at the apex of testes. Control, *n* = 17; Tj > eIF5 RNAi, *n* = 20; Tj > eIF5 OE; eIF5 RNAi, *n* = 11. **D** The number of Zfh1-positive cells at the apex of testes. Control, *n* = 17; Tj > eIF5 RNAi, *n* = 20; Tj > eIF5 OE; eIF5 RNAi, *n* = 11. **E** The number of pointed fusomes at the apex of testes. Control, *n* = 12; Tj > eIF5 RNAi, *n* = 11; Tj > eIF5 OE; eIF5 RNAi, *n* = 5. **F** Immunostaining of Vasa (red) and 1B1 (green) at the apex of control, Tj > eIF5 RNAi, and Tj > eIF5 OE; eIF5 RNAi testes. DNA was stained with Hoechst (blue). (**P < 0.01; ***P < 0.001, n.s. indicates non-significant. Scale bar: 50 μm)

First of all, we analyzed the testicular eIF5 expression pattern via single nucleus RNA sequencing (snRNA-seq) data [28] and found that eIF5 was widely expressed in *Drosophila* testes, especially in germ cells and cyst cells (Additional file 1: Fig. S1). To investigate the function of eIF5 in CySC lineage, we used Tj-GAL4 to manipulate the levels of *eIF5* in *Drosophila* testes. We next detected the knockdown efficiency using quantitative real-time reverse transcription PCR (qRT-PCR) and found that the relative mRNA level of *eIF5* was reduced in Tj > eIF5 RNA interference (RNAi) testes when compared with those in the controls (Fig. 1A). To further characterize the eIF5 loss-of-function assay, the eIF5 protein in *Drosophila* testes was analyzed by using a mass spectrometry (MS)-based targeted quantitative proteomic method in parallel reaction monitoring (PRM) mode [29, 30]. Three different specific heavy peptides (LQDLTDGA**K**, SVTDIFY**R**, and VNTFIV**K**) of eIF5 were synthesized and spiked into tryptic peptides of *Drosophila* testes. The results revealed that the eIF5 protein expression level was dramatically reduced in Tj > eIF5 RNAi testes compared with control testes (Additional file 1: Fig. S2).

Next, we asked whether translation initiation factor eIF5 affected cyst cell division. Therefore, we stained for cyst markers to distinguish cyst cells at different periods. Zinc-finger homeodomain protein 1 (Zfh1) was used as a marker for CySCs surrounding hub cells, while Eyes absent (Eya) was typically observed in mature cyst cells [31, 32]. Interestingly, the Eya signal was not detected (Fig. 1B, C), while Zfh1-positive cyst cells dramatically increased in Tj > eIF5 RNAi testes compared with those in the controls (Fig. 1B, D), indicating that dysfunctional cyst cells, mediated by the lack of eIF5, induced excessive accumulation of CySCs and led to the loss of mature cyst cells.

Previously, CySCs were proven to be essential for germ cell differentiation in *Drosophila* testes [22, 33, 34]. We next used 1B1, a fusome marker for dynamic changes in morphology, to observe the differentiation process of germ cells in *Drosophila* testes. In the Tj > eIF5 RNAi testes, undifferentiated germ cells accumulated and the number of pointed fusomes increased significantly compared with those in the controls (Fig. 1E, F), which blocked germ cell differentiation and eventually generated extra GSC-like masses in the testes.

Next, we used a rescue assay (Tj > eIF5 overexpression (OE); eIF5 RNAi) to observe whether the eIF5 RNAi-mediated phenotype could be rescued in *Drosophila* testes. By dissection (Additional file 1: Fig. S3), we found that about 83.3% (*n* = 162) of eIF5 RNAi testes driven by Tj-GAL4 lacked testicular morphological structures and this situation could be partially reversed in Tj > eIF5 OE; eIF5 RNAi testes (43.0% testes lacking morphological structures, *n* = 100). Among testes with morphological structures, we observed that the number of CySCs and mature cyst cells could be recovered in Tj > eIF5 OE; eIF5 RNAi testes (Fig. 1B, D). Importantly, germ cell differentiation defects, which were mediated by knocking down *eIF5*, could also be rescued in Tj > eIF5 OE; eIF5 RNAi testes (Fig. 1E, F).

### eIF5 deficiency mediated cyst cell differentiation and non-cell autonomous effects of germ cell differentiation in adult testes

**Fig. 2:**
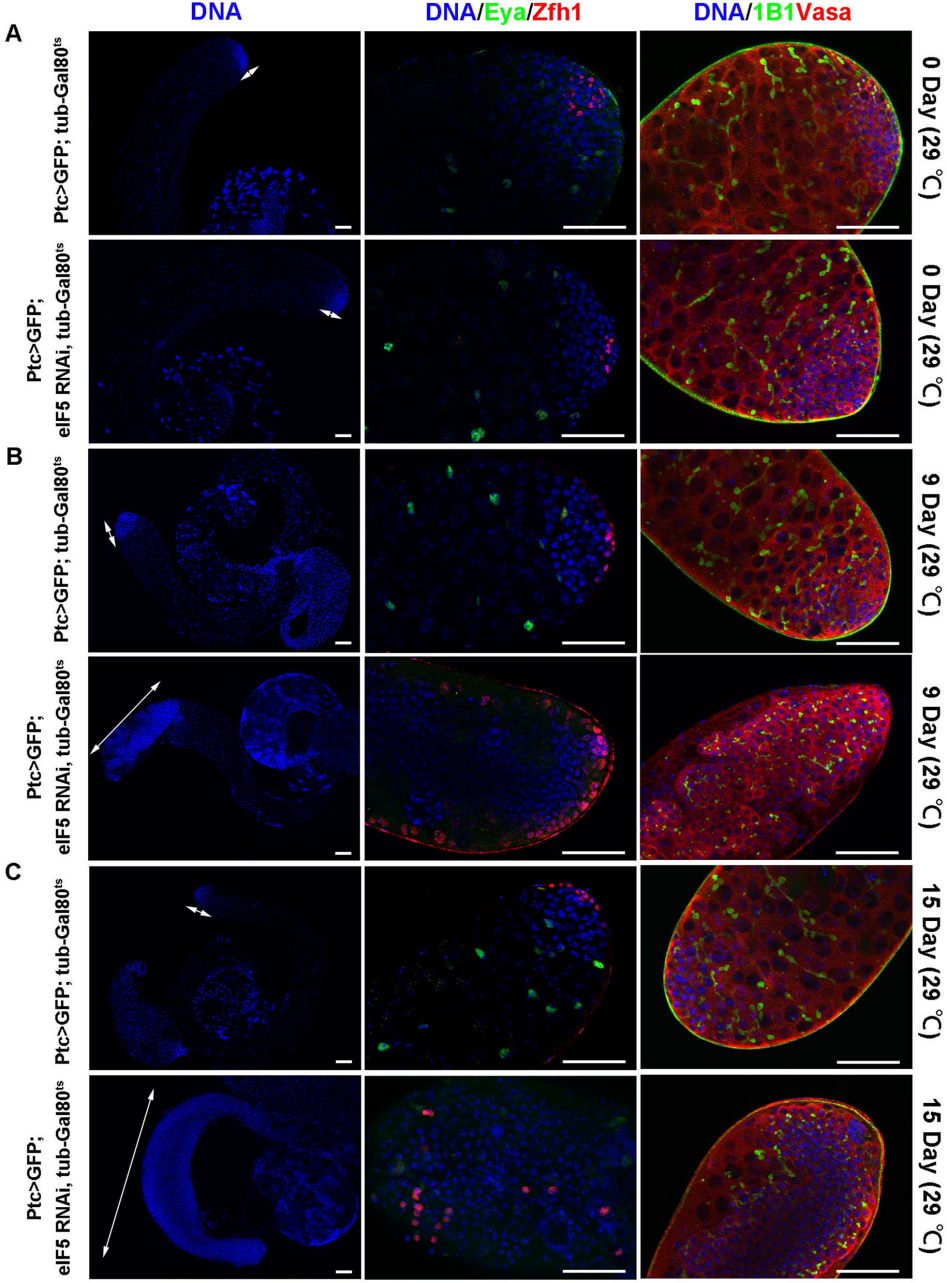
Somatic eIF5 is required for cyst cell differentiation and non-autonomously affects germ cell differentiation in adult testes. **A** Immunostaining of Vasa (red), 1B1 (green), Zfh1(red), and Eya (green) at the apex of testes of Ptc > GFP; tub-Gal80^ts^ and Ptc > GFP; eIF5 RNAi, tub-Gal80^ts^ at 0 days (29 °C). **B** Immunostaining of Vasa (red), 1B1 (green), Zfh1(red), and Eya (green) at the apex of testes of Ptc > GFP; tub-Gal80^ts^ and Ptc > GFP; eIF5 RNAi, tub-Gal80^ts^ at 9 days (29 °C). **C** Immunostaining of Vasa (red), 1B1 (green), Zfh1(red), and Eya (green) at the apex of testes of Ptc > GFP; tub-Gal80^ts^ and Ptc > GFP; eIF5 RNAi, tub-Gal80^ts^ at 15 days (29 °C). White double arrows represented regions of undifferentiated cells. DNA was stained with Hoechst (blue). Scale bars: 50 µm

Reducing the *eIF5* level using Tj-Gal4 led to the formation of tumorlike structures, and both cyst cell and germ cell differentiation were defective. To investigate whether a lack of eIF5 could block cyst cell and germ cell differentiation at the adult stage, we manipulated *eIF5* expression at the early stage of cyst cells driven by Ptc-Gal4, UAS-GFP; tub-Gal80^ts^ fly strain. Flies with genotype of Ptc > GFP; tub-Gal80^ts^ and Ptc > GFP; eIF5 RNAi, tub-Gal80^ts^ were cultured at the 18 °C until enclosure, and then the culture temperature was shifted to 29 °C and testes were analyzed at 0, 9, and 15 days. GFP signal could not be detected at 0 day (29 °C) but was detected at the apex of the testes at 9 and 15 days (29 °C) (Additional file 1: Fig. S4). No obvious abnormality of cyst cells and germ cells was found at 0 day (29 °C) (Fig. 2A). However, the accumulation of undifferentiated cyst cells and germ cells was observed and mature cyst cells were totally lost in testes at 9 and 15 days (29 °C) after knockdown of *eIF5* by Ptc-Gal4 (Fig. 2B, C). Taken together, these results suggested that eIF5 could regulate cyst cell differentiation and then provide non-autonomous signals for germ cell differentiation in adult testes.

### CySC lineage deficient for eIF5 displays aberrant cell proliferation and restricts germ cell differentiation at the mitotic stage

**Fig. 3:**
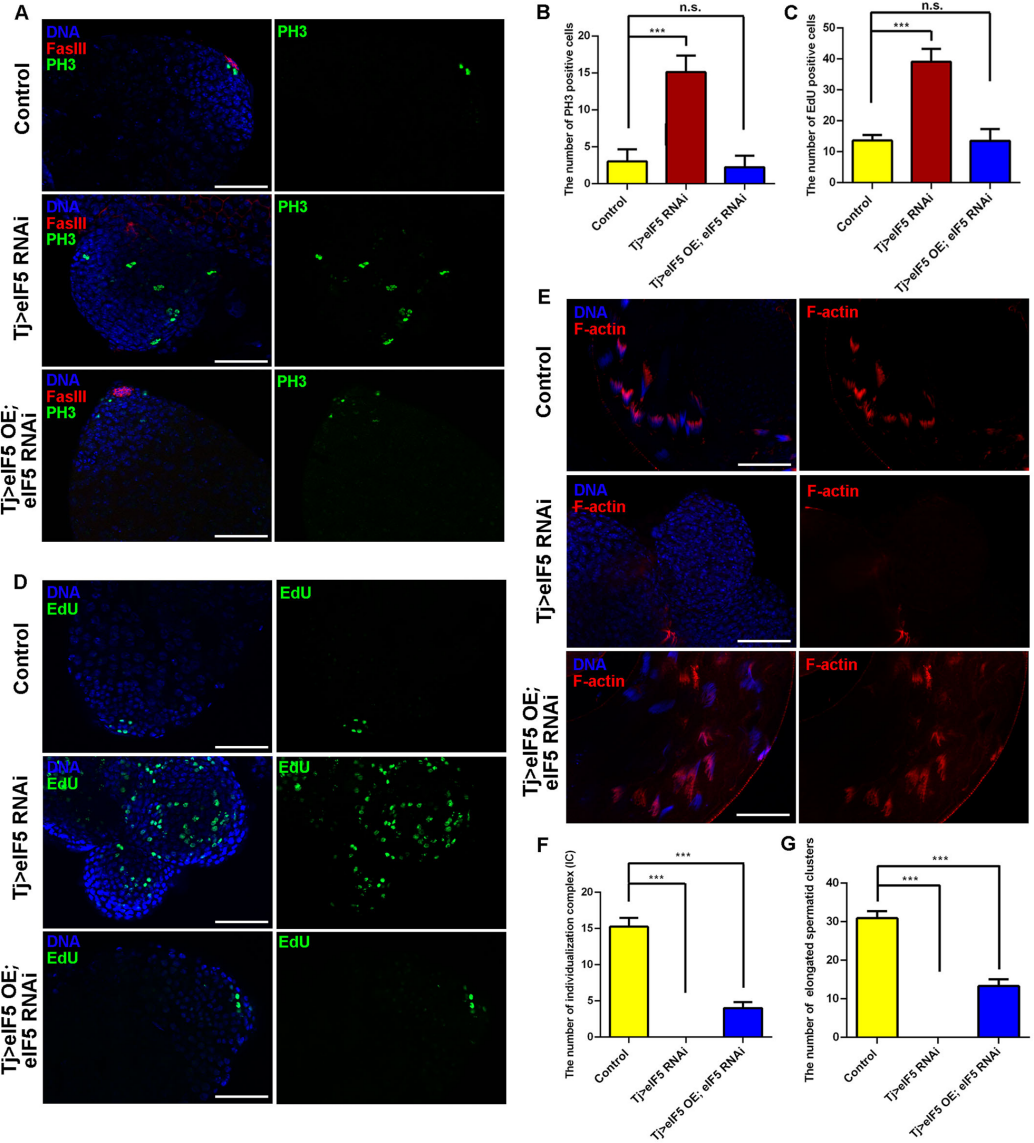
eIF5 deficiency in CySCs mediated hyperproliferation and affected spermatogenesis. **A** Immunostaining of PH3 (green) and FasIII (red) at the apex of control, Tj > eIF5 RNAi, and Tj > eIF5 OE; eIF5 RNAi testes. **B** The number of PH3-positive cells at the apex of testes. Control, *n* = 12; Tj > eIF5 RNAi, *n* = 9; Tj > eIF5 OE; eIF5 RNAi, *n* = 9. **C** The number of EdU-positive cells at the apex of testes. Control, *n* = 11; Tj > eIF5 RNAi, *n* = 11; Tj > eIF5 OE; eIF5 RNAi, *n* = 8. **D** EdU staining (green) at the apex of control, Tj > eIF5 RNAi, and Tj > eIF5 OE; eIF5 RNAi testes. **E** F-actin staining (red) of control, Tj > eIF5 RNAi, and Tj > eIF5 OE; eIF5 RNAi testicular tails. **F** The number of individualization complexes (ICs) in testicular tails. Control, *n* = 12; Tj > eIF5 RNAi, *n* = 11; Tj > eIF5 OE; eIF5 RNAi, *n* = 12. **G** The number of elongated spermatid clusters in testicular tails. Control, *n* = 12; Tj > eIF5 RNAi, *n* = 11; Tj > eIF5 OE; eIF5 RNAi, *n* = 12. DNA was stained with Hoechst (blue). (***P < 0.001. n.s. indicates nonsignificant. Scale bars: 50 µm)

In Tj > eIF5 RNAi testes, no hub signal (no FasIII-positive signal) existed among these tumorlike structures, indicating a lack of regulation of stem cell niche signals (Fig. 3A). To further characterize the undifferentiated cell populations, we observed that the number of phospho-histone H3 (PH3)-positive cells, which represent mitotic cells at the division phase, increased dramatically in Tj > eIF5 RNAi testes compared with those in the controls (Fig. 3A, B). We also examined the number of cells in S phase by counting the number that incorporated 5-ethynyl-2′-deoxyuridine (EdU). As expected, the number of EdU-positive cells also increased in Tj > eIF5 RNAi testes compared with those in the controls (Fig. 3C, D). Moreover, both PH3-positive and EdU-positive cells could be recovered in Tj > eIF5 OE; eIF5 RNAi testes, compared with those in the controls (Fig. 3A–D).

Cyclin A is expressed throughout premeiotic G2 phase and coordinates the synchronized divisions of germ cells via intercellular fusome bridges [35]. Cyclin B is normally downregulated at the end of the mitotic divisions and is not detectable in early spermatocytes [36]. We next examined these two phase-specific cell cycle markers (Cyclin A and Cyclin B) in control and Tj > eIF5 RNAi testes. Among testes tumorlike structures, Cyclin A signals could be detected in Tj > eIF5 RNAi testes (Additional file 1: Fig. S5A), and spermatogonia-expressed Cyclin B was highly activated in GSC-like masses in Tj > eIF5 RNAi testes (Additional file 1: Fig. S5B). These results suggested that deficiency of eIF5 in the CySC lineage led to proliferation disorders without normal stem cell niche signals and might disturb the cell cycle during spermatogenesis.

To further assess the degree of disruption of germ cell differentiation, we stained the cells with several markers for the late stage of germ cells. Previous reports suggested that oo18 RNA-binding protein (Orb) was highly enriched in elongated spermatids undergoing nuclear condensation [37, 38]. In the present study, we also found low expression levels of Orb in mitotic spermatogonia and a high expression level in elongated spermatids in the control testes (Additional file 1: Fig. S5C). However, Orb-positive elongated spermatids were totally lost, while Orb levels were upregulated in GSC-like masses in Tj > eIF5 RNAi testes (Additional file 1: Fig. S5C). We also found that the individualization complex (IC) and elongated spermatid clusters were totally lost in Tj > eIF5 RNAi testes and could be partially rescued in Tj > eIF5 OE; eIF5 RNAi testes (Fig. 3E–G). These data indicated that accumulated GSC-like masses, induced by eIF5 deficiency in CySCs, were blocked at the mitotic stage during spermatogenesis.

### Ectopic expression of eIF5 in CySC lineage does not affect the stem cell niche regulation in *Drosophila* testes

To evaluate the effect of overexpressing *eIF5* in the stem cell niche in *Drosophila* testes, we generated UAS-3xHA-eIF5 CDS transgenic flies and drove *eIF5* expression in CySCs using Tj-Gal4. First, the HA-eIF5 fusion protein could be detected in Tj > eIF5 OE testes (Additional file 1: Fig. S6A). Next, we analyzed cyst cell and germ cell characteristics systemically. There was a dramatic increase in Eya-positive cyst cells in Tj > eIF5 OE testes compared with those in the controls (Additional file 1: Fig. S6B, C). However, the number of Zfh1-positive cyst cells displayed no significant difference after overexpressing *eIF5* in CySCs (Additional file 1: Fig. S6B, D). We also observed that germ cells displayed no obvious differentiation disruption in Tj > eIF5 OE testes compared with that in the controls (Additional file 1: Fig. S6E, F).

Furthermore, we detected whether proliferation was affected by the ectopic expression of eIF5 in cyst cells. We found that the number of PH3-positive cells and EdU-positive cells was unchanged in Tj > eIF5 OE testes compared with those in the controls (Additional file 1: Fig. S7A–D). Moreover, the number of IC and elongated spermatid clusters decreased after overexpressing *eIF5* in CySCs (Additional file 1: Fig. S7E–G); however, they were not enough to cause germ cell differentiation defects. These observations suggested that dysfunctions of Zfh1-positive cyst cells might be responsible for the germ cell differentiation defects.

### eIF5 inhibits cell death in vivo and in vitro

**Fig. 4:**
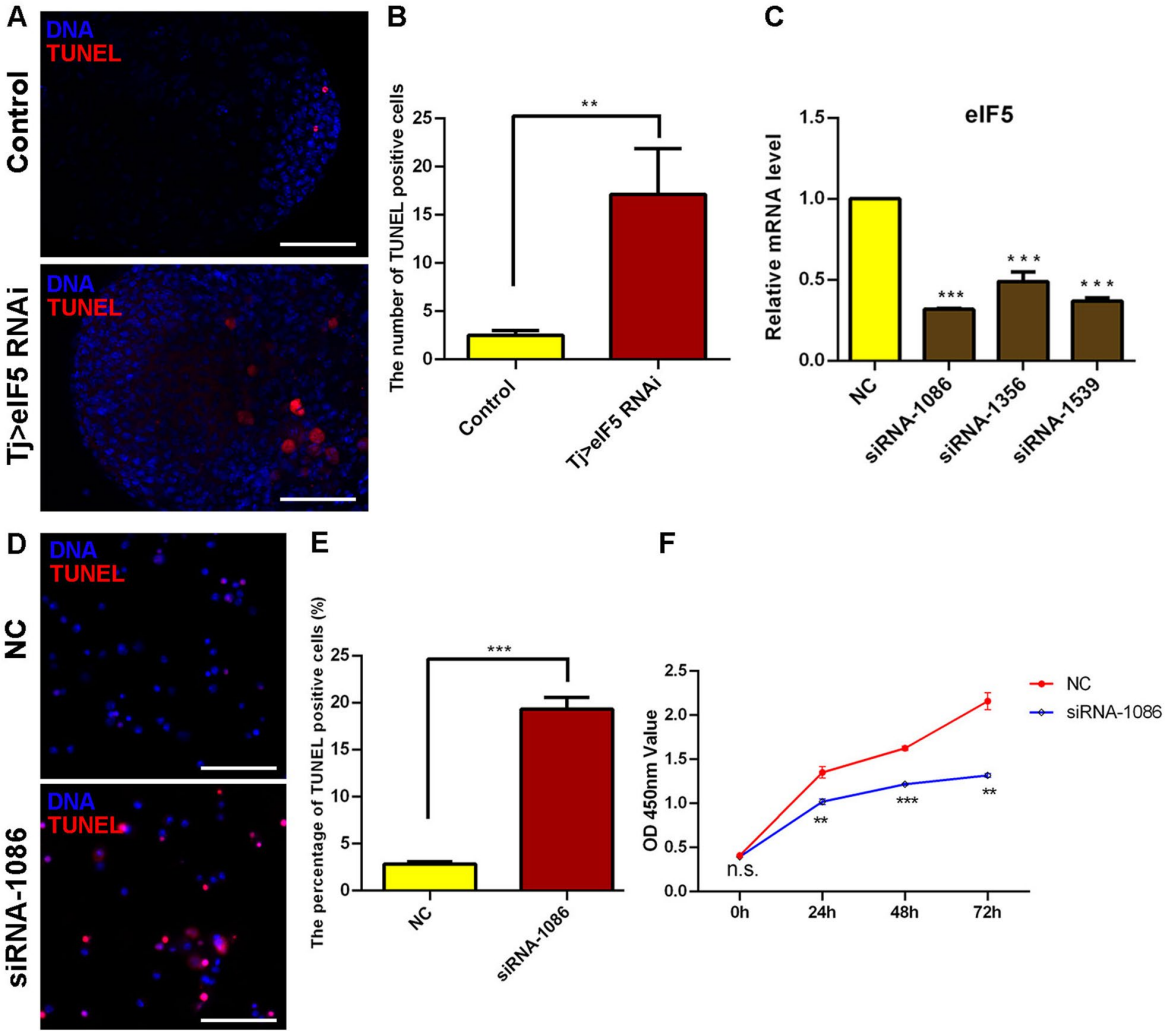
Reduction in eIF5 increased cell apoptosis. **A** TUNEL staining at the apex of control and Tj > eIF5 RNAi testes. **B** The number of TUNEL-positive cells at the apex of testes. Control, *n* = 14; Tj > eIF5 RNAi, *n* = 15. **C** Relative mRNA level of *eIF5* in S2 cells treated with NC, siRNA-1086, siRNA-1356, and siRNA-1539. **D** TUNEL staining in S2 cells treated with NC and siRNA-1086. **E** The percentage of TUNEL-positive cells in S2 cells. NC, *n* = 10; siRNA-1086, *n* = 9. **F** CCK-8 assay for S2 cells transfected with NC and siRNA-1086. *n* = 3 per group. DNA was stained with Hoechst (blue). (**P < 0.01; ***P < 0.001. Scale bars: 50 µm)

To determine whether eIF5 mediates cell survival, we stained cells with terminal deoxynucleotidyl transferase-mediated dUTP-biotin nick-end labeling (TUNEL) via in vivo and in vitro approaches. In Tj > eIF5 RNAi testes, the number of TUNEL-positive cells increased markedly compared with those in the controls, indicating increased cell death among GSC-like masses mediated by silencing *eIF5* in CySCs (Fig. 4A, B). However, we observed no obvious change in cell death in the Tj > eIF5 OE testes (Additional file 1: Fig. S8).

We further knocked down *eIF5* by treating S2 cells with three small interfering RNAs (siRNAs) (siRNA-1086, siRNA-1356, and siRNA-1539). siRNA-1086 had the best interference efficiency and was thus used for subsequent cytological experiments (Fig. 4C). We found that the number of TUNEL-positive cells increased dramatically in siRNA-1086-treated S2 cells compared with that in the negative control (NC) group (Fig. 4D, E). Moreover, we also used a Cell Counting Kit-8 (CCK-8) assay to construct a cell growth curve, and siRNA-1086-treated S2 cells showed an inhibited growth ability (Fig. 4F). Taken together, these data indicated that eIF5 could regulate cell death in both *Drosophila* testes and S2 cells.

### Transcriptional regulation of eIF5 in S2 cells

**Fig. 5:**
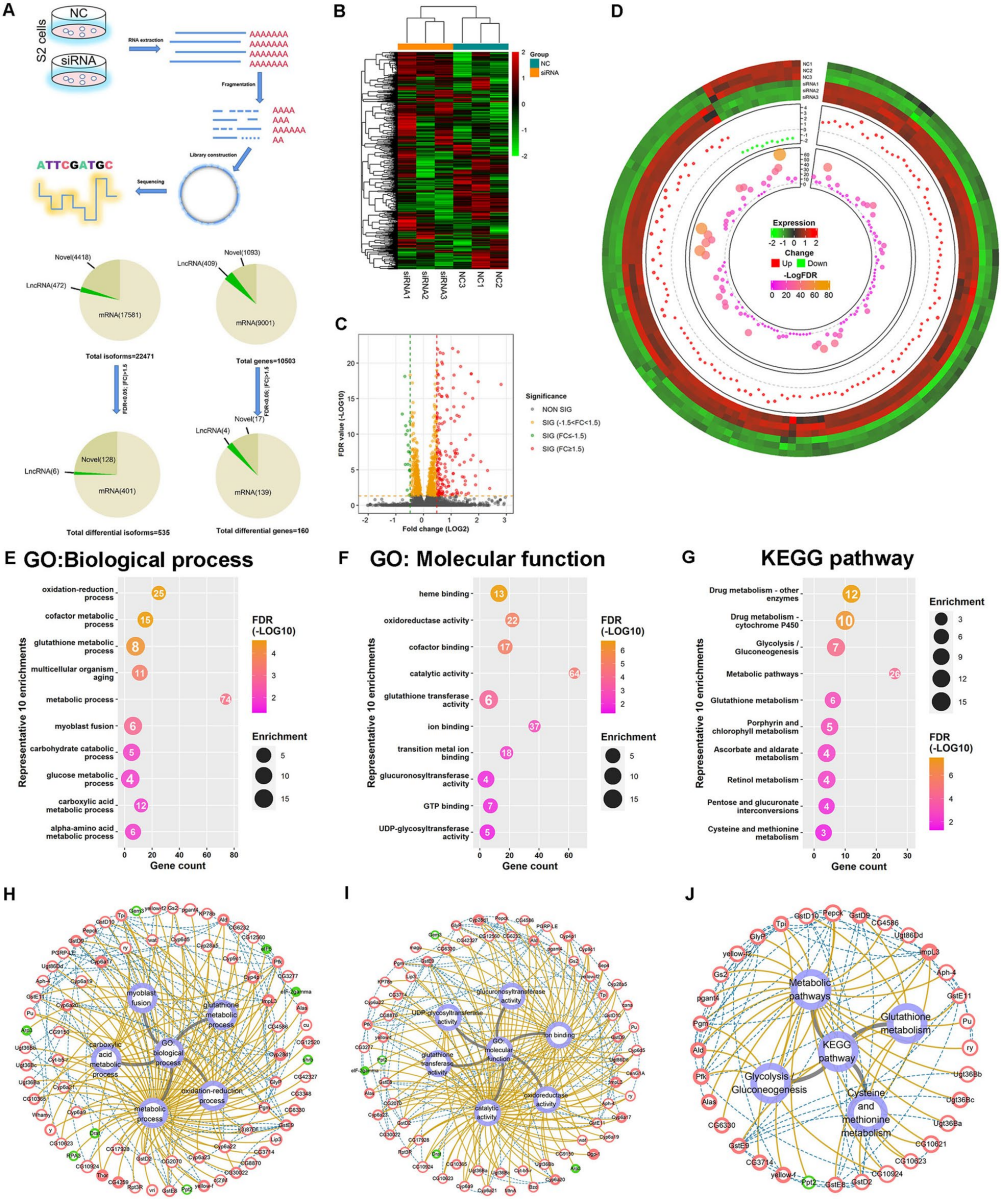
Transcription profiling of eIF5 in S2 cells. **A** Schematic diagram of the RNA-seq experiment in S2 cells. **B** Clustering heatmap of genes from the comparison between the NC and siRNA-1086 groups by Pearson correlation. The expression values were further normalized using the Z-score method. Red and green indicate high and low expression values, respectively. **C** Volcano plots based on -log_10_FDR and log_2_FC from the comparison of the NC and siRNA-1086 groups. **D** Circos plot of differentially expressed genes from the comparison of the NC and siRNA-1086 groups. From outer to inner, the tracks are expression heatmap, dots for FC, and dots for -logFDR values correspondingly. **E–G** Ten representative significantly enriched terms of GO: Biological process (**E**), GO: Molecular function (**F**) and KEGG pathways (**G**). Scaled colors correspond to -log_10_FDR values, while the circle size is in proportion to the enrichment value. The number in each circle indicates the gene count. **H–J** Interaction network of selected genes and their associations with GO: Biological process (**H**), GO: Molecular function (**I**) and KEGG pathways (**J**). Red and green colors represent up- and downregulation, respectively. The overall size and border size are in proportion to the FC and − log_10_FDR values correspondingly. Blue dashed lines and brown solid lines indicate gene-to-gene interactions and gene-to-function relationships, respectively

Since some eIFs have been revealed selective roles for gene transcription via multiple signals [39, 40], we performed RNA sequencing (RNA-seq) analysis in NC and *eIF5* siRNA-treated S2 cells to further explore the regulatory network of eIF5. In the transcriptional sequence profiles, 22,471 isoforms and 10,503 genes were detected. We then analyzed differentially expressed isoforms and genes (false discover rate (FDR) < 0.05; |fold change (FC)| > 1.5), and identified 535 isoforms corresponding to 160 differentially expressed genes, including 143 upregulated genes and 17 downregulated genes in S2 cells silenced for *eIF5* (Fig. 5A and Additional file 5: Table S4, Additional file 6: Table S5). Heatmap views revealed the overall gene expression, and Volcano plot views also displayed distinct differences between NC and *eIF5* siRNA-treated S2 cells (Fig. 5B, C). Moreover, Circos plot views displayed the integrity distributions of the expression values, FC, and FDR values for the differentially expressed genes in S2 cells (Fig. 5D).

To further analyze eIF5 functions in S2 cells, gene ontology (GO) and Kyoto Encyclopedia of Genes and Genomes (KEGG) pathway analysis were performed for eIF5-associated transcriptional network in S2 cells. Among the biological processes (Fig. 5E), eIF5-associated differentially expressed genes were involved in multiple metabolic processes (e.g., oxidation–reduction process, cofactor metabolic process, glutathione metabolic process, and glucose metabolic process). GO analysis for molecular function also revealed that catalytic activity, ion binding, oxidoreductase activity, cofactor binding, glutathione transferase activity, GTP binding, and glucuronosyltransferase activity might participate in the regulation of eIF5 in S2 cells (Fig. 5F). Moreover, KEGG pathway analysis revealed enrichments in metabolic pathways, drug metabolism, glycolysis/gluconeogenesis, and glutathione metabolism (Fig. 5G). Complex gene relation network analysis was further used to refine and construct gene-to-gene interactions, gene expression changes, and their associations with biological process, molecular function, and KEGG pathways (Fig. 5H–J). Based on the gene relation network analysis, we noticed that genes encoding several proteins, e.g., translation initiation factors eIF2γ, Ldh (also called ImpL3), and Glutathione S transferase subunits (e.g., GstD2, GstD10, and GstE11), were identified to play key roles in the eIF5-associated transcriptional network. Taken together, our data suggested that eIF5-associated transcriptional cofactors participated in multiple metabolic processes, which might result in cell death of S2 cells.

**Fig. 6:**
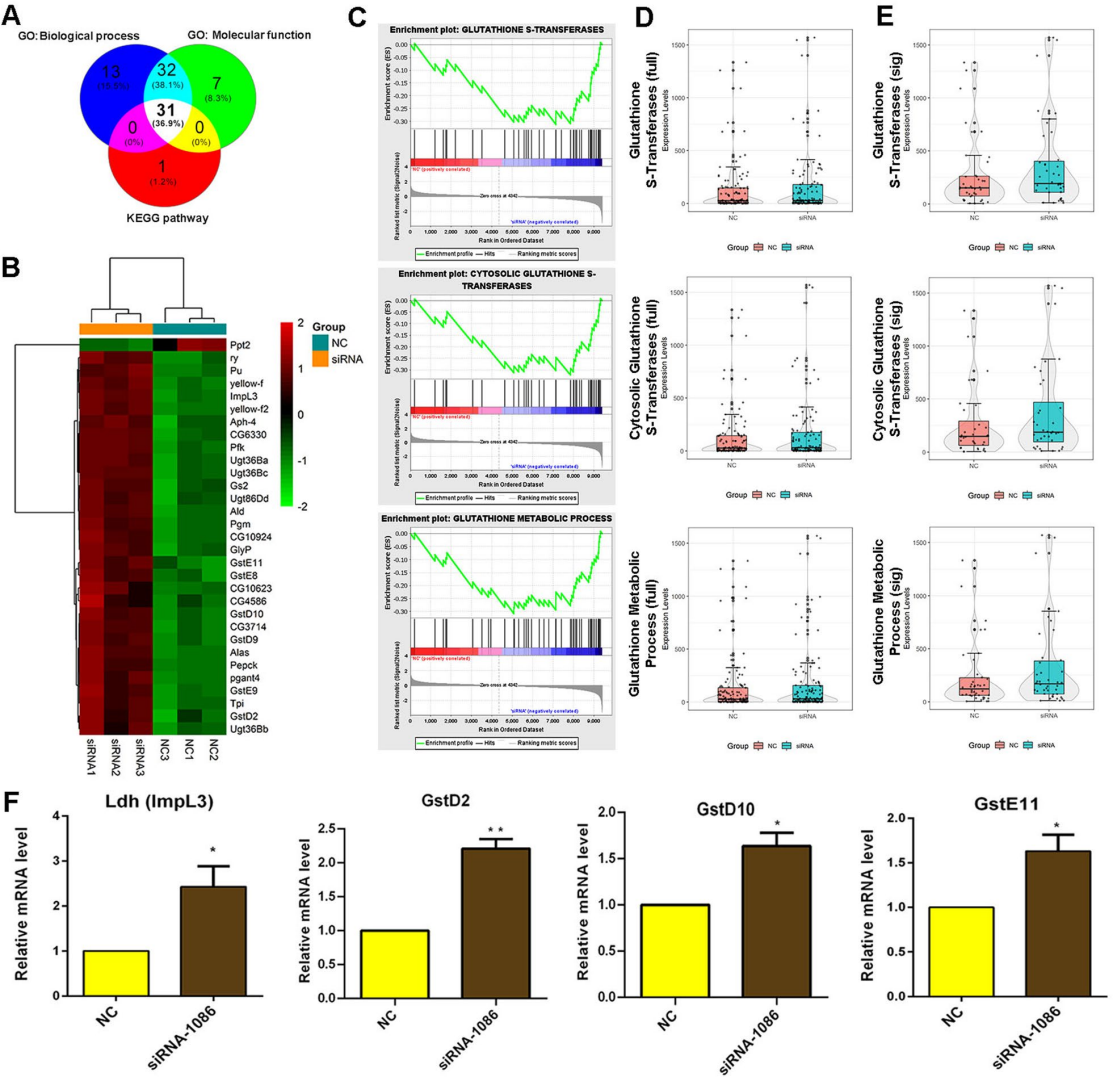
Integrated analysis of the eIF5-mediated regulatory network and key factors in S2 cells. **A** Venn diagram of the integrated analysis of biological process, molecular function, and KEGG pathways. **B** Clustering heatmap of 31 common elements that were hits in biological process, molecular function, and KEGG pathways. **C** Enrichment plot of glutathione S-transferases, cytosolic glutathione S-transferases, and glutathione metabolic process. **D** Violin plot analysis of glutathione S-transferases, cytosolic glutathione S-transferases, and glutathione metabolic process for all identified genes. **E** Violin plot analysis of glutathione S-transferases, cytosolic glutathione S-transferases, and glutathione metabolic process for differentially expressed genes. **F** Relative mRNA level of representative genes in the NC and siRNA-1086-treated S2 cells. (*P < 0.05; **P < 0.01)

To further verify our conclusions, integrated analysis of the RNA-seq data identified 31 common elements that were hits in all three analyses (biological process, molecular function, and KEGG pathway) (Fig. 6A). Among these common elements, heatmap views showed that almost all the genes were upregulated in *eIF5* siRNA-treated S2 cells (Fig. 6B). In addition, we used the overall expression trend for in-depth and comprehensive gene function enrichment analysis by gene set enrichment analysis (GSEA) with statistical criteria of *P* < 0.05, FDR < 0.25, |normalized enrichment score (NES)|> 1. Interestingly, we identified that enrichment plots of glutathione S-transferases, cytosolic glutathione S-transferases, and glutathione metabolic process tended to be enriched and highly expressed in the *eIF5* siRNA group (Fig. 6C). Meanwhile, box-violin plot views of overall expression trends for all detected genes did not display significant changes, while overall expression trends for differentially expressed genes were obviously upregulated for glutathione S-transferases, cytosolic glutathione S-transferases, and glutathione metabolic process (Fig. 6D, E). We next verified the relative mRNA levels of representative eIF5-associated cofactors, which might regulate oxidative stress homeostasis and cell fate functions. We found that *Ldh* (*Impl3*), *GstD2*, *GstD10*, and *GstE11* were upregulated in *eIF5* siRNA-treated S2 cells compared with those in the NC group, which was consistent with the transcriptome profiling (Fig. 6F). These data deepened our understanding of eIF5 function in cell fate and provided important clues to explain how a lack of eIF5 induced the formation of TGCTs.

### CySC lineage eIF5 mediates cyst cell and germ cell differentiation with eIF1A and eIF2γ

**Fig. 7:**
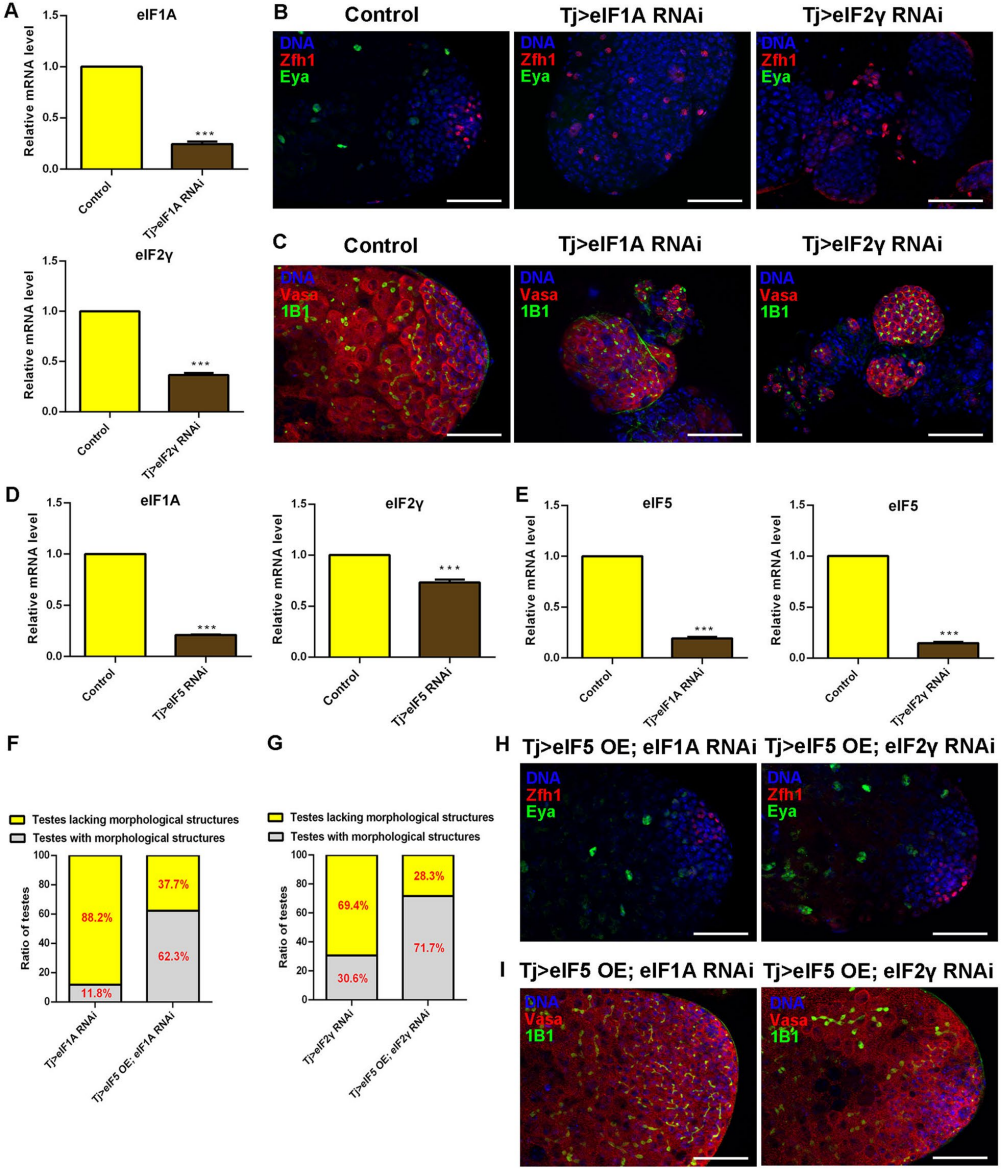
CySC lineage eIF5 regulates eIF1A and eIF2γ in *Drosophila* testes. **A** Knockdown efficiency of *eIF1A* and *eIF2γ* in control and corresponding RNAi testes, respectively. *n* = 3 per group. **B** Immunostaining of Zfh1 (red) and Eya (green) at the apex of control, Tj > eIF1A RNAi, and Tj > eIF2γ RNAi testes. **C** Immunostaining of Vasa (red) and 1B1(green) at the apex of control, Tj > eIF1A RNAi, and Tj > eIF2γ RNAi testes. **D** Relative mRNA level of *eIF1A* and *eIF2γ* in control and Tj > eIF5 RNAi testes. *n* = 3 per group. **E** Relative mRNA level of *eIF5* in control, Tj > eIF1A RNAi, and Tj > eIF2γ RNAi testes. *n* = 3 per group. **F** Proportion of testes lacking morphological structures in Tj > eIF1A RNAi and Tj > eIF5 OE; eIF1A RNAi testes. Tj > eIF1A RNAi, *n* = 136; Tj > eIF5 OE; eIF1A RNAi, *n* = 61. **G** Proportion of testes lacking morphological structures in Tj > eIF2γ RNAi and Tj > eIF5 OE; eIF2γ RNAi testes. Tj > eIF2γ RNAi, *n* = 124; Tj > eIF5 OE; eIF2γ, RNAi *n* = 53. **H** Immunostaining of Zfh1 (red) and Eya (green) at the apex of Tj > eIF5 OE; eIF1A RNAi and Tj > eIF5 OE; eIF2γ RNAi testes. **I** Immunostaining of Vasa (red) and 1B1(green) at the apex of Tj > eIF5 OE; eIF1A RNAi and Tj > eIF5 OE; eIF2γ RNAi testes. DNA was stained with Hoechst (blue). (***P < 0.001. Scale bars: 50 µm)

We questioned whether other translation initiation factors are also involved in the formation of TGCTs in testes. eIF5, eIF1A, and eIF2γ participate in the assembly of the pre-initiation complex [16]. Therefore, we tested whether eIF1A and eIF2γ were associated with similar phenotypes of cell differentiation in testes. We knocked down *eIF1A* and *eIF2γ* expression in CySCs, separately (Fig. 7A). We found that CySCs accumulated and mature cyst cells were totally lost in both Tj > eIF1A RNAi and Tj > eIF2γ RNAi testes (Fig. 7B). Moreover, we also demonstrated that undifferentiated germ cells with pointed fusome accumulated in *eIF1A* RNAi and *eIF2γ* RNAi testes driven by Tj-Gal4 (Fig. 7C). These data showed that knockdown of eIF1A and eIF2γ mimicked the phenotype of knockdown of eIF5 in the stem cell niche in *Drosophila* testes.

According to the transcriptional network in S2 cells, eIF5 could regulate the expression level of the key components of the pre-initiation complex. We next confirmed whether the expression level of *eIF1A* and *eIF2γ* could be affected in testes. Interestingly, both *eIF1A* and *eIF2γ* mRNA expression levels were downregulated dramatically in Tj > eIF5 RNAi testes (Fig. 7D). Moreover, we also found that eIF5 levels decreased dramatically in both Tj > eIF1A RNAi and Tj > eIF2γ RNAi testes (Fig. 7E). To further explore the potential synergistic activities between eIF5 and eIF1A (or eIF2γ), we tested their genetic interactions. We examined whether ectopic expression of eIF5 could rescue the phenotype of testes tumorlike structures induced by silencing of *eIF1A* or *eIF2γ* in CySCs. Firstly, we found about 88.2% (*n* = 136) of Tj > eIF1A RNAi testes lacked morphological structures, and the proportion was dramatically reduced to 37.7% (*n* = 61) in Tj > eIF5 OE; eIF1A RNAi testes (Fig. 7F). Similarly (Fig. 7G), the proportion of testes lacking morphological structures decreased dramatically from 69.4% (*n* = 124, Tj > eIF2γ RNAi) to 28.3% (*n* = 53, Tj > eIF5 OE; eIF2γ RNAi). In the genetic interaction analysis, we showed that CySCs and mature cyst cells patterns could be rescued in Tj > eIF5 OE; eIF1A RNAi and Tj > eIF5 OE; eIF2γ RNAi testes (Fig. 7H). Moreover, the germ cell differentiation process and Orb-enriched elongated spermatids could also be recovered by the ectopic expression of eIF5 in both Tj > eIF1A RNAi and Tj > eIF2γ RNAi testes (Fig. 7I and Additional file 1: Fig. S9). Our data strongly suggested that eIF5, eIF1A, and eIF2γ prevent the formation of TGCTs by cyst cell and germ cell differentiation via the pre-initiation complex.

## Discussion

**Fig. 8:**
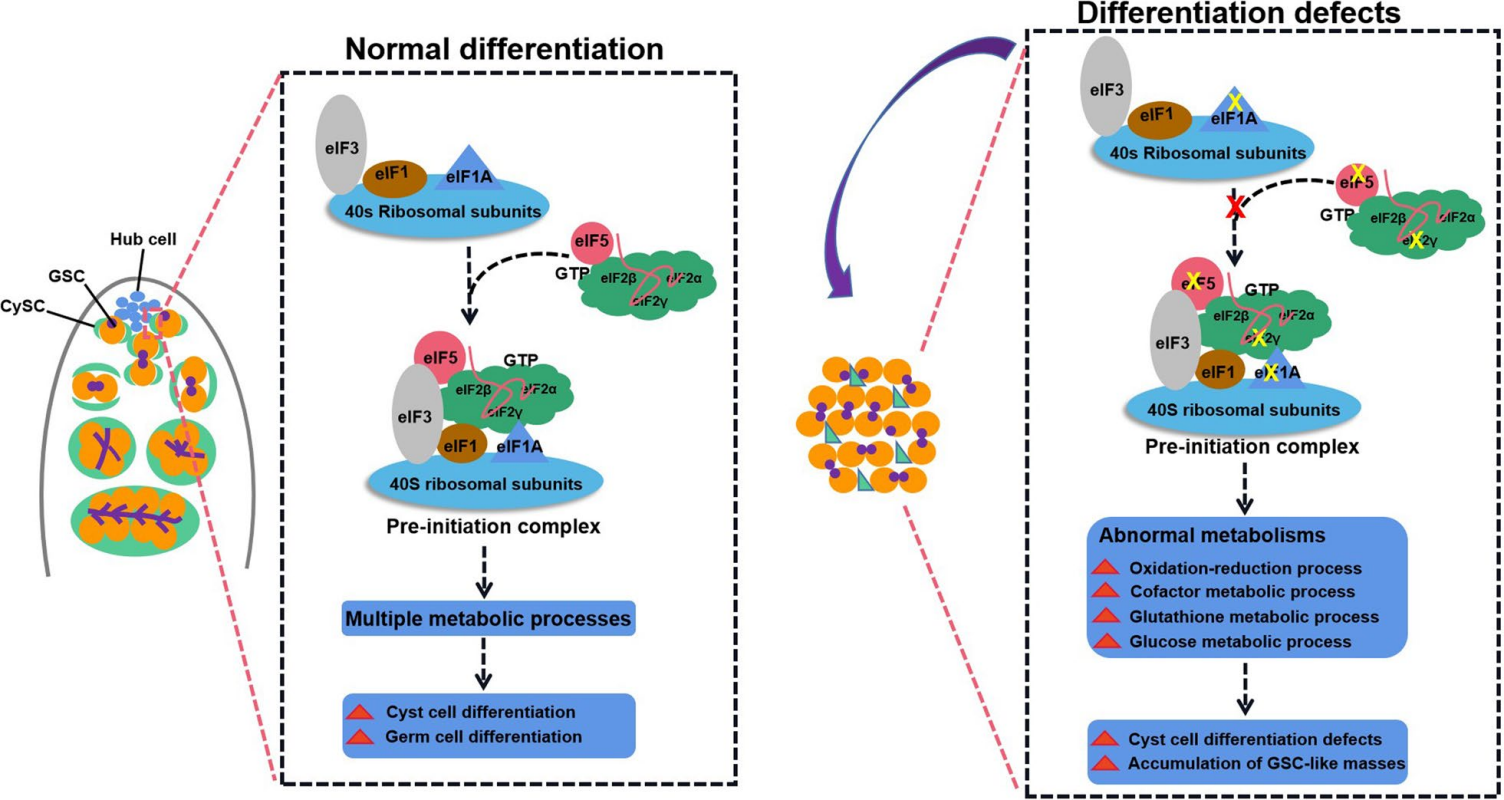
Schematic diagram of the pre-initiation complex in the male stem cell niche. This study provided a model for the formation of TGCTs via the pre-initiation complex in testes. In the male stem cell niche of *Drosophila* testes, the pre-initiation complex in the CySC lineage mediated normal cyst cell and germ cell differentiation via multiple metabolic processes. However, CySCs lacking eIF5, eIF1A, or eIF2γ, which destroyed the assembly of the pre-initiation complex, had abnormal metabolism (e.g., oxidation–reduction process, cofactor metabolic process, glutathione metabolic process, and glucose metabolic process), leading to cyst cell differentiation defects and the accumulation of GSC-like masses

In *Drosophila* testes, the stem cell niche is studied as a model to mimic the formation of TGCTs. *Drosophila* provides a powerful genetically tractable system to study how germ cell differentiation has adapted in response to changes in external signals in testes [41–43]. Our study investigated roles of translation initiation factor eIF5 in *Drosophila* testes systemically and uncovered that CySC lineage eIF5 was essential for cyst cell differentiation, which then promoted germ cell differentiation via non-autonomous cell effects (Fig. 8). In Tj > eIF5 RNAi testes, cyst cells were arrested at the CySC stage, while the accumulated GSC-like masses were blocked at mitotic spermatogonia stage. eIF5 deficiency also induced an increase in cell death in vivo and in vitro. We considered these might be caused by disrupting multiple metabolic and cell cycle processes.

eIF5, eIF1A, and eIF2γ are key elements for translation initiation and participate in the assembly of the pre-initiation complex. Interestingly, eIF1A and eIF2γ mimicked the phenotype of eIF5 in *Drosophila* testes. In the genetic interaction analysis, ectopic expression of eIF5 in *eIF1A* RNAi or *eIF2γ* RNAi testes was sufficient to reverse the testicular phenotype of differentiation defects, which indicated potential therapeutic targets for the treatment of TGCTs. Meanwhile, we also noticed that CySCs lacking these translation initiation factors had germ cell differentiation defects without normal hub signals, suggesting that the eIF5, eIF1A, and eIF2γ-formed pre-initiation complex in CySCs also has the ability to alter hub cell fate in *Drosophila* testes.

Soma–germline communications within the stem cell niche are highly conserved and are essential for the regulation of the homeostasis of cell proliferation and differentiation in the gonad [44]. Studies have illustrated that soma–germline signals play instructive roles in controlling stem cell fate during spermatogenesis [45, 46]. Normal signals from the stem cell niche guide the transition from proliferation to meiotic differentiation, which is especially important for development into terminally differentiated germ cells and fertility [45]. Without instructive signals originating from CySCs, the early-stage germ cells fail to initiate differentiation and hyperproliferative GSC-like masses accumulate in *Drosophila* testes [47].

Secreted signals in somatic cells within the stem cell niche, e.g., BMP and epidermal growth factor receptor (EGFR) signaling pathways, have been used to mediate soma–germline communications. Somatic Thickveins (Tkv) promoted germ cell differentiation via Smad-independent signals to restrict the activation of the BMP signaling pathway within the stem cell niche [48]. Recently, mRNA splicing factor CG6015 was revealed to be required for the regulation of stem cell niche signals and could mediate germ cell differentiation via the EGFR signaling pathway in *Drosophila* testes [33]. EGFR signaling is considered a canonical signaling pathway for the regulation of soma–germline interactions. Overexpression of dominant negative forms of *Ras oncogene at 85D* (*Ras85d*) and *Egfr* in the soma induced large increases in primordial germ cells (PGCs), indicating that EGFR signaling also functions in somatic support cells to regulate PGC divisions in *Drosophila* ovaries [10]. Stem cell tumor (Stet) could also guide GSC differentiation via proper connections between somatic and germline cells in both testes and ovaries [49]. Importantly, the chromatin factor Enhancer of Polycomb [E(Pc)] acts in cyst cells and is responsible for germline differentiation and germ cell fate maintenance via negative regulation of transcription-related genes in multiple signaling pathways [50]. Meanwhile, evidence suggested that ecdysone signaling regulates early-stage germ cell differentiation via soma–germline interactions by modulating cell adhesion-mediated steroid-let-7-Wingless signaling [51, 52]. These studies emphasized the significance of niche signals, where germ cells reside, in antagonizing CySC lineage identity and promoting germ cell differentiation, and provided a model in which CySC lineage signals strictly control soma–germline communications in response to external stimuli.

eIF3 forms the main frame structure for the large multiprotein complex to promote the binding of eIFs to the 40S ribosomal subunit. More importantly, the binding of eIF1 and eIF5 further strengthens the compact architecture of eIF3 [53]. Moreover, eIF5 has GTPase activity, which together with eIF2 promotes AUG selection more strictly and binds to the 40S ribosomal subunit at the vacated position of eIF1 to facilitate the recognition of the start codon [54]. eIF5 also has other regulatory functions within the eIF5/eIF2•GDP complex, which are independent of its GTPase activating protein (GAP) function, such as preventing the spontaneous release of GDP from eIF2, thus acting as a GDP dissociation inhibitor (GDI) [55, 56]. Overexpression of *eIF5* can inhibit the formation of the multiprotein complex, and the eIF2/eIF5 complex antagonizes guanine nucleotide exchange for coordinated regulation of translation initiation [57].

Previous studies have shown that CySC lineage cells lacking small ribosomal subunits, e.g., ribosomal protein S13 (RpS13), ribosomal protein S26 (RpS26), ribosomal protein S30 (RpS30), ribosomal protein S15Aa (RpS15Aa), and ribosomal protein S15Ab (RpS15Ab), lead to the accumulation of undifferentiated GSC-like masses [14, 43]. In this study, we found that cyst cells lacking eIF5, eIF1A, and eIF2γ, which are key components of the pre-initiation complex, mimicked the phenotype of cells lacking several small ribosomal subunits for germ cell differentiation. Together, these data suggested that the pre-initiation complex might regulate cyst cell and germ cell differentiation directly, thereby contributing to the prevention of TGCTs.

## Conclusions

In conclusion, we explored the roles of eIF5 via the pre-initiation complex for the regulation of the stem cell niche in *Drosophila* testes. Our study revealed that translation initiation factor eIF5, together with its co-regulators eIF1A and eIF2γ, acted in the CySC lineage to mediate both cyst cell and germ cell differentiation and maintained the balance of proliferation and cell death. Further investigation of the mechanisms of translation initiation in the stem cell niche might provide new insights and develop strategies to treat TGCTs.

## Supplementary Information

**Additional file 1.** Supplementary materials.

**Additional file 2.** Identified isoforms in eIF5-associated transcriptional profiling.

**Additional file 3.** Identified genes in eIF5-associated transcriptional profiling.

**Additional file 4.** Detailed information of the siRNAs used in this study.

**Additional file 5.** Primer sequences for qRT-PCR.

**Additional file 6.** Antibodies used in this study.

## Data Availability

The original contributions presented in the study are included in the article/Supplementary Material; further inquiries can be directed to the corresponding author/s.
