# Supplementary material for: Cyst stem cell lineage eIF5 non-autonomously prevents testicular germ cell tumor formation via eIF1A/eIF2γ-mediated pre-initiation complex
Source: Stem Cell Res Ther. 2022 Jul 26;13:351. doi: 10.1186/s13287-022-03025-5 (PMC9327282; doi:10.1186/s13287-022-03025-5)
Supplement: Supplementary file 1 — Additional file 1. Supplementary materials. [file 13287_2022_3025_MOESM1_ESM.doc]

**Supplementary Materials**


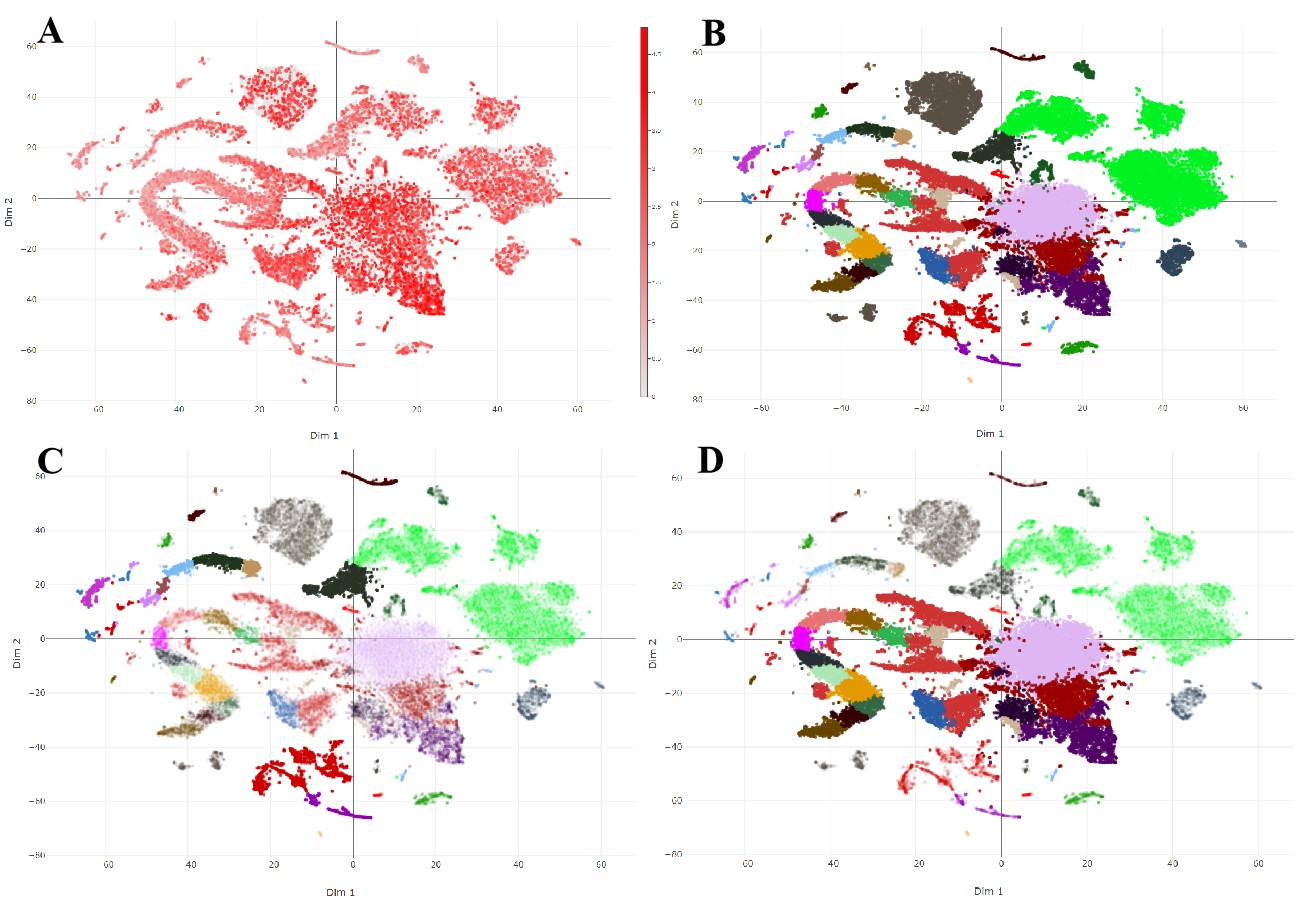


**Supplementary Figure 1. eIF5 expression pattern analysis by snRNA-seq.** (A) eIF5 expression pattern in *Drosophila* testes. (B) Cell clusters in *Drosophila* testes. (C) Cyst cell clusters in *Drosophila* testes (Dark labeled cell populations). (D) Germ cell clusters in *Drosophila* testes (Dark labeled cell populations). Data were accessed from a single-nucleus transcriptomic atlas of the adult fruit fly (Fly Cell Atlas; www.flycellatlas.org).

**
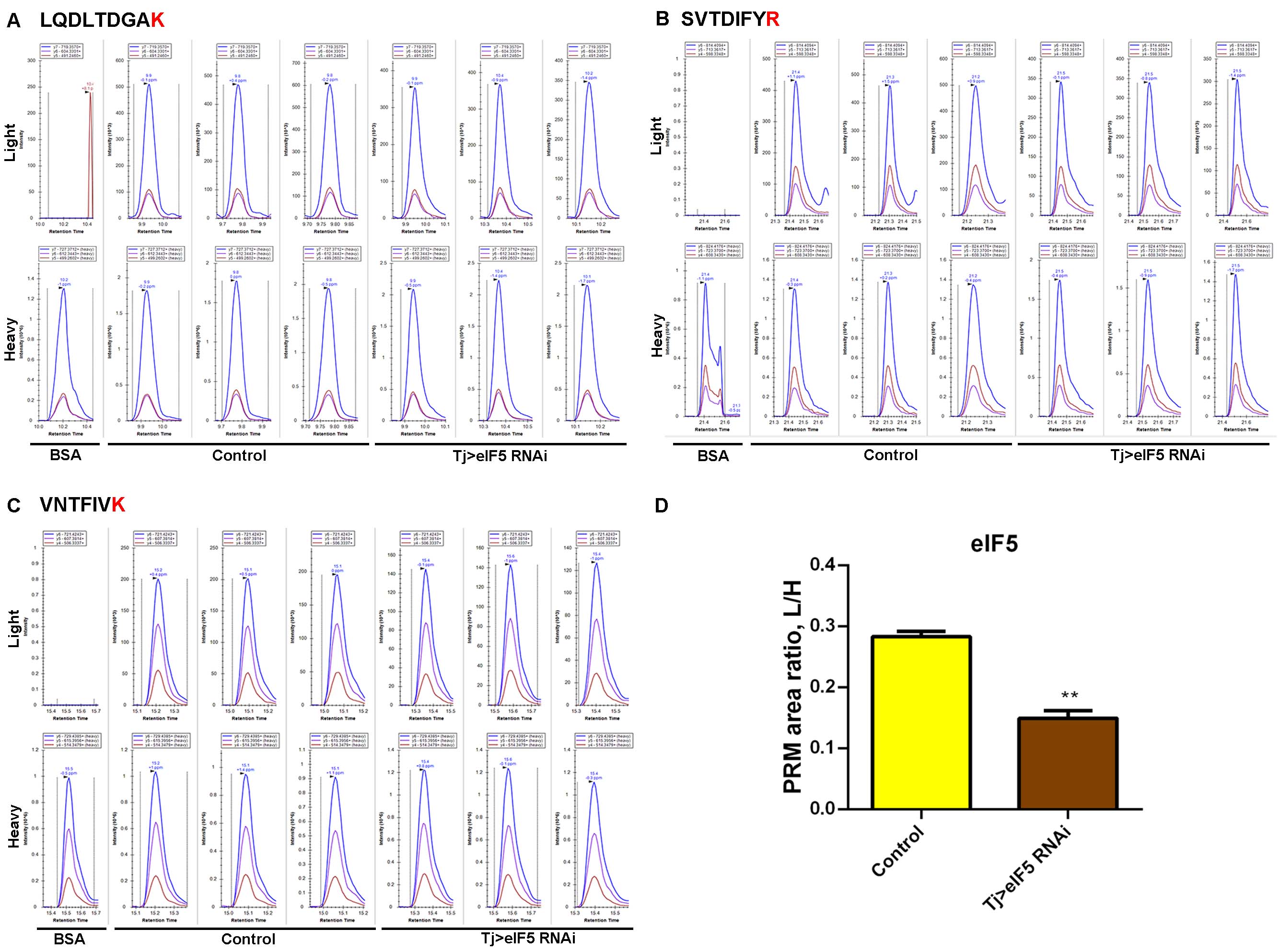
**

**Supplementary Figure 2. Validation of eIF5 protein expressions in control and Tj>eIF5 RNAi testes by PRM-MS.** (A-C) LQDLTDGA**K**, SVTDIFY**R**, and VNTFIV**K** peptides derived from eIF5 protein along with internal heavy standards were detected. Heavy peptides with BSA, isotope-labeled peptides were spiked into tryptic peptides of control (n=3) and Tj>eIF5 RNAi (n=3) testes in *Drosophila*. (D) Relative quantification of eIF5 protein expression in control and Tj>eIF5 RNAi testes. L-light peptide, H-heavy peptide; the eIF5 protein level was quantified as the PRM area ratio of L/H peptides. (***P* < 0.01).

**
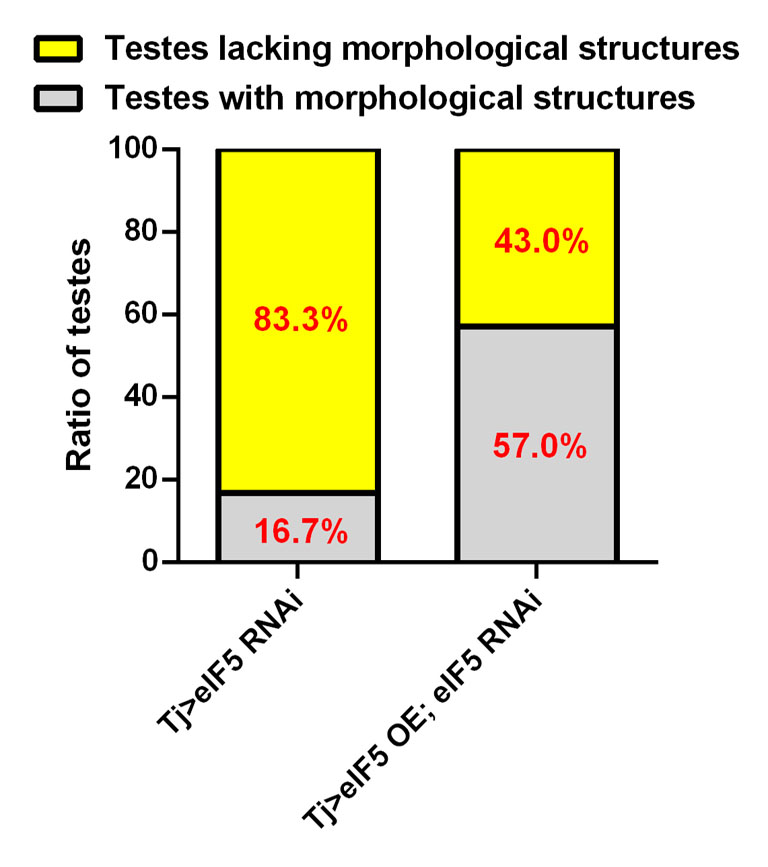
**

**Supplementary Figure 3.** **Ratio of testes lacking morphological structures in Tj>eIF5 RNAi and Tj>eIF5 OE; eIF5 RNAi testes.** Tj>eIF5 RNAi, n=162; Tj>eIF5 OE; eIF5 RNAi, n=100.


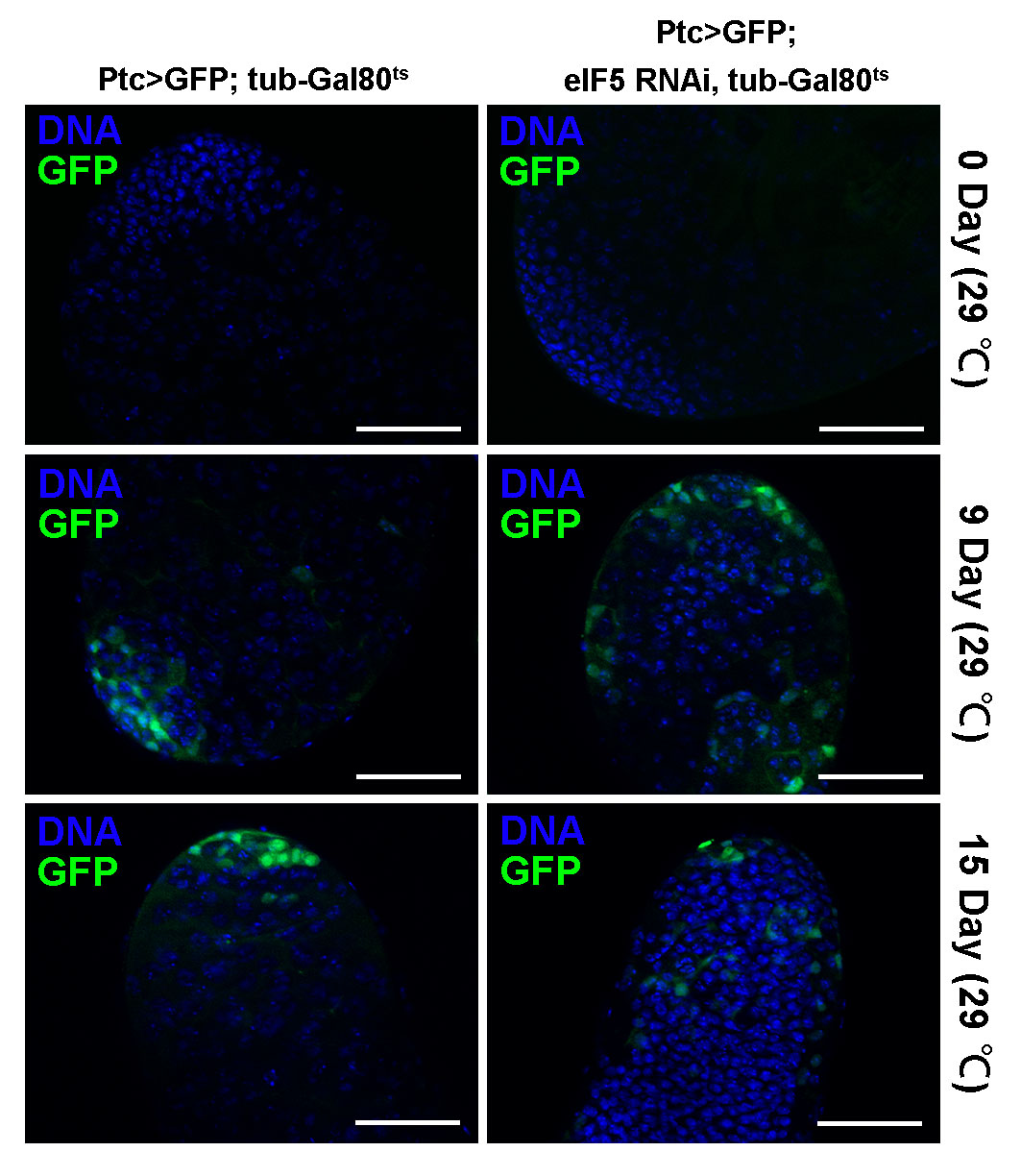


**Supplementary Figure 4. GFP expression pattern driven by Ptc-Gal4 in adult testes.** GFP (green) expression pattern at the apex of Ptc>GFP;tub-Gal80^ts^ and Ptc>GFP; eIF5 RNAi, tub-Gal80^ts^ testes at 0 days (29 °C), 9 days (29 °C) and 15 days (29 °C). DNA was stained with Hoechst (blue). Scale bars: 50 µm.

**
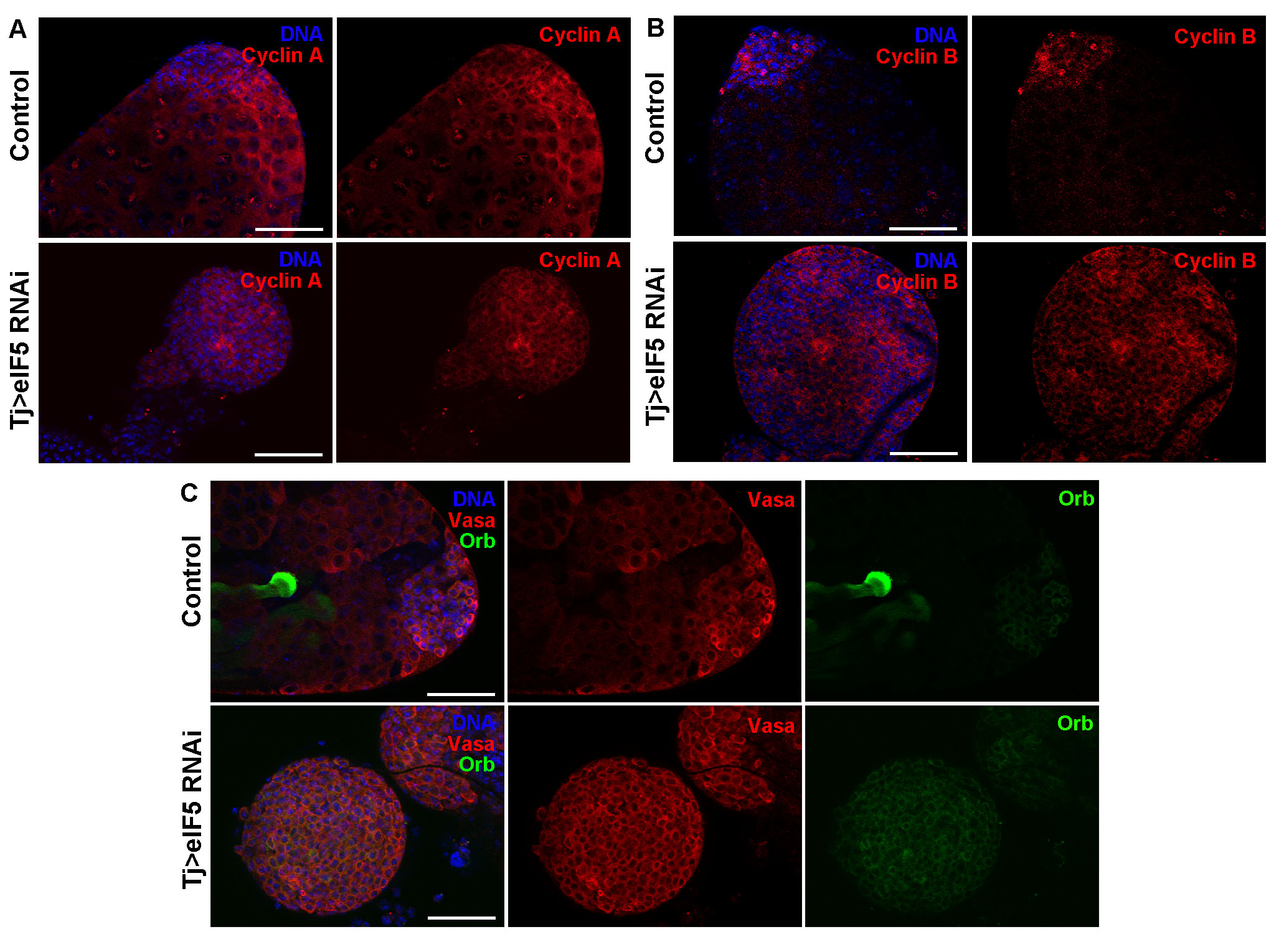
**

**Supplementary Figure 5. Germ cell differentiation was blocked at mitotic spermatogonia by eIF5 deficiency in CySC lineage.** (**A**) Immunostaining of Cyclin A (red) at the apex of control and Tj>eIF5 RNAi testes. (**B**) Immunostaining of Cyclin B (red) at the apex of control and Tj>eIF5 RNAi testes. (**C**) Immunostaining of Vasa (red) and Orb (green) at the apex of control and Tj>eIF5 RNAi testes. DNA was stained with Hoechst (blue). Scale bars: 50 µm.


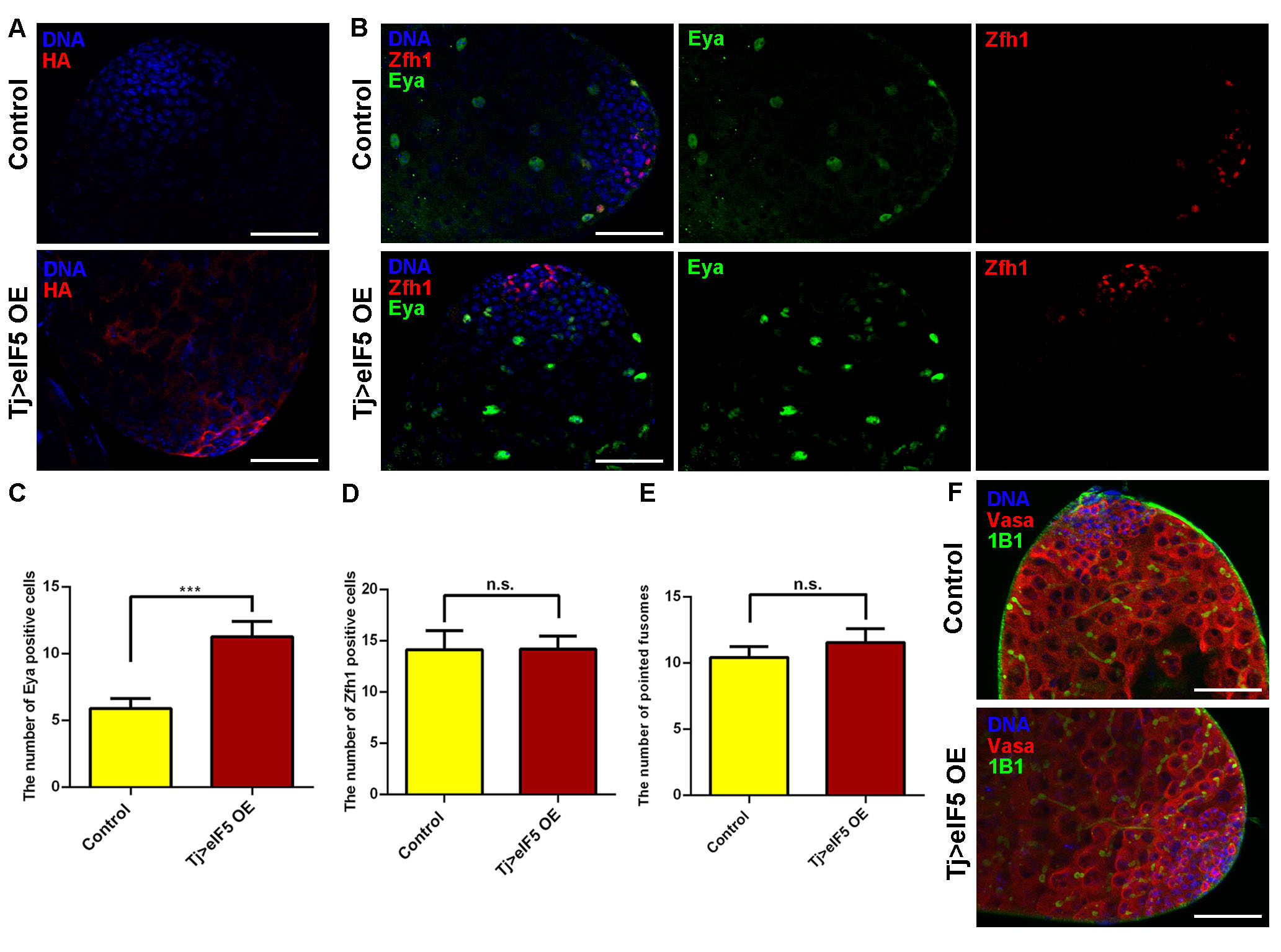


**Supplementary Figure 6. Ectopic expression of eIF5 in CySC lineage did not influence cyst cell characteristics and germ cell differentiation in *Drosophila* testes.** (**A**) Immunostaining of HA (red) at the apex of control and Tj>eIF5 OE testes. (**B**) Immunostaining of Zfh1 (red) and Eya (green) at the apex of control and Tj>eIF5 OE testes. (**C**) The number of Eya-positive cells at the apex of testes. Control, n=17; Tj>eIF5 OE, n=12). (**D**) The number of Zfh1‑positive cells at the apex of testes. Control, n=17; Tj>eIF5 OE, n=12. (**E**) The number of pointed fusomes at the apex of testes. Control, n=12; Tj>eIF5 OE, n=11. (**F**) Immunostaining of Vasa (red) and 1B1 (green) at the apex of control and Tj>eIF5 OE testes. DNA was stained with Hoechst (blue). (****P* < 0.001. n.s. indicates non-significant. Scale bars: 50 µm).

**
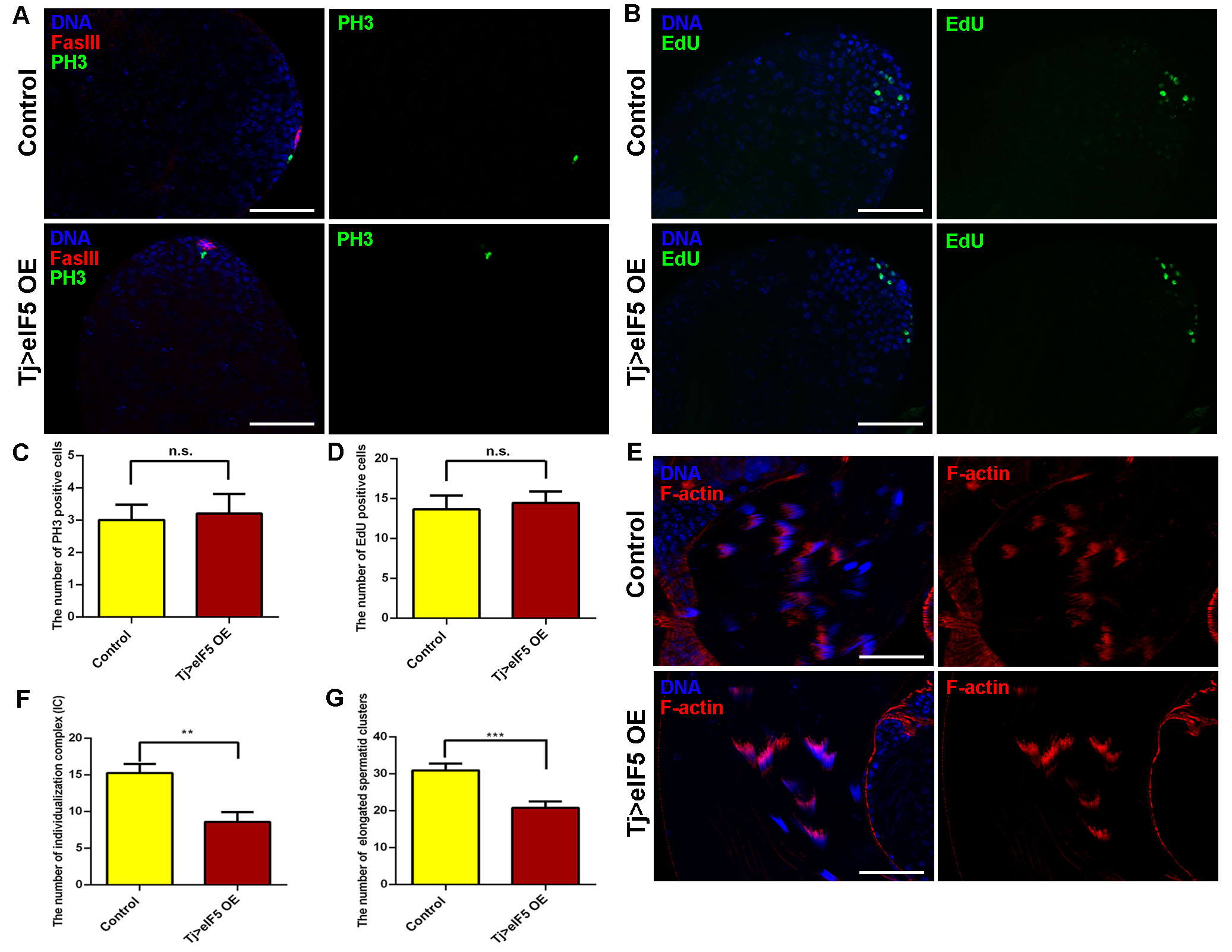
**

**Supplementary Figure 7. Ectopic expression of eIF5 in CySC lineage did not affect proliferation.** (**A**) Immunostaining of FasⅢ (red) and PH3 (green) at the apex of control and Tj>eIF5 OE testes. (**B**) EdU staining at the apex of control and Tj>eIF5 OE testes. **(C)** The number of PH3-positive cells at the apex of testes.Control, n=12; Tj>eIF5 OE, n=10. (**D**) The number of EdU-positive cells at the apex of testes.Control, n=11; Tj>eIF5 OE, n=11. (**E**) F-actin staining of control and Tj>eIF5 OE testicular tails. (**F**) The number of individualization complex (IC) in testicular tails.Control, n=12; Tj>eIF5 OE, n=12. (**G**) The number of elongated spermatid clusters in testicular tails. Control, n=12; Tj>eIF5 OE, n=12. DNA was stained with Hoechst (blue). (***P* < 0.01; ****P* < 0.001. n.s. indicates non-significant. Scale bars: 50 µm).


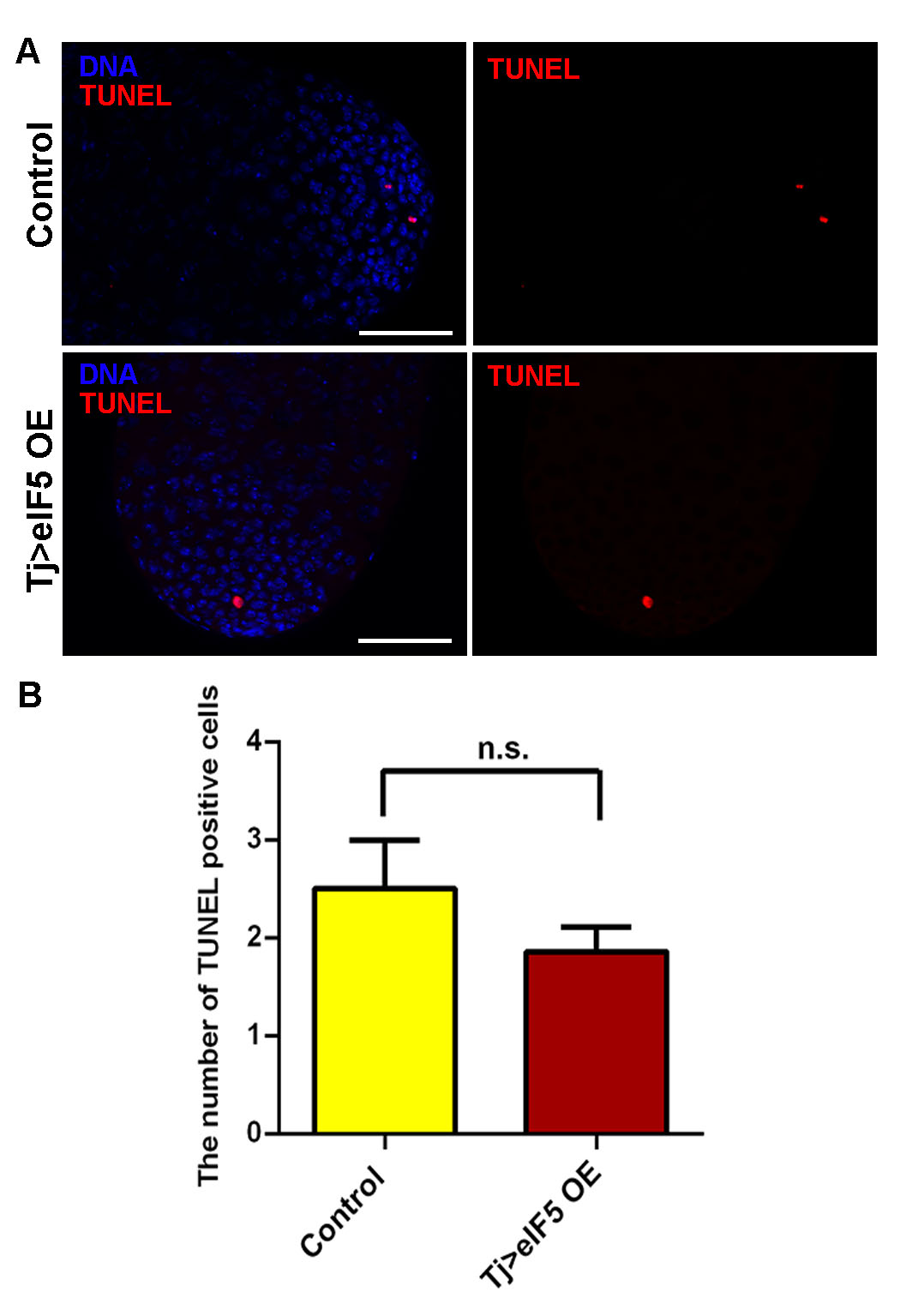


**Supplementary Figure 8. Ectopic expression of eIF5 in the CySC lineage did not affect cell apoptosis.** (**A**) TUNEL staining at the apex of control and Tj>eIF5 OE testes. (**B**) The number of TUNEL-positive cells at the apex of testes. Control, n=14; Tj>eIF5 OE, n=14. DNA was stained with Hoechst (blue). (n.s. indicates non-significant. Scale bars: 50 µm).


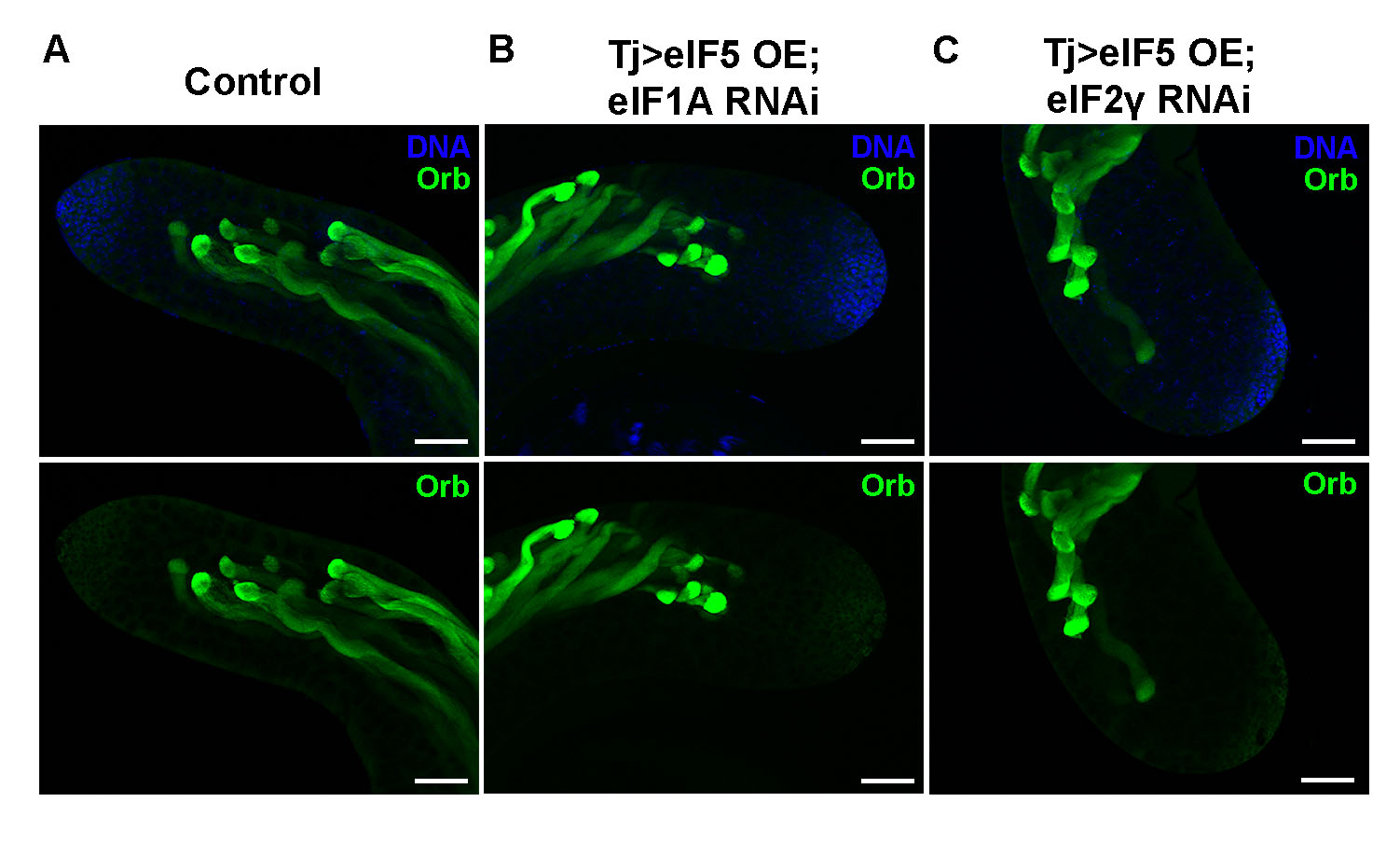


**Supplementary Figure 9. Ectopic expression of eIF5 in the CySC lineage was sufficient to restore elongated spermatid formation in *eIF1A* and *eIF2γ* RNAi testes.** Immunostaining of Orb (green) at the apex of control (**A**), Tj>eIF5 OE; eIF1A RNAi (**B**) and Tj>eIF5 OE; eIF2γ RNAi (**C**) testes. DNA was stained with Hoechst (blue). Scale bars: 50 µm.

**Supplementary Table 1. Identified isoforms in eIF5-associated transcriptional profiling.**

**Supplementary Table 2. Identified genes in eIF5-associated transcriptional profiling.**

**Supplementary Table 3. Detailed information of the siRNAs used in this study.**

**Supplementary Table 4. Primer sequences for qRT-PCR.**

**Supplementary Table 5. Antibodies used in this study.**
