# Supplementary material for: Cyst stem cell lineage eIF5 non-autonomously prevents testicular germ cell tumor formation via eIF1A/eIF2γ-mediated pre-initiation complex
Source: Stem Cell Res Ther. 2022 Jul 26;13:351. doi: 10.1186/s13287-022-03025-5 (PMC9327282; doi:10.1186/s13287-022-03025-5)
Supplement: Supplementary file 4 — Additional file 4. Detailed information of the siRNAs used in this study. [file 13287_2022_3025_MOESM4_ESM.doc]

**Table S3. Detailed information of the siRNAs used in this study.**

| **siRNA** | **Sense (5’-3’)** | **Antisense (5’-3’)** |
| --- | --- | --- |
| NC | UUCUCCGAACGUGUCACGUTT | ACGUGACACGUUCGGAGAATT |
| eIF5 siRNA-1086 | GCAAAGGAAACGGCAUUAATT | UUAAUGCCGUUUCCUUUGCTT |
| eIF5 siRNA-1356 | CCAUUUCGCAGUCUUGCAATT | UUGCAAGACUGCGAAAUGGTT |
| eIF5 siRNA-1539 | CCAAUAACUCUUUGGCCAATT | UUGGCCAAAGAGUUAUUGGTT |
