# Supplementary material for: Cyst stem cell lineage eIF5 non-autonomously prevents testicular germ cell tumor formation via eIF1A/eIF2γ-mediated pre-initiation complex
Source: Stem Cell Res Ther. 2022 Jul 26;13:351. doi: 10.1186/s13287-022-03025-5 (PMC9327282; doi:10.1186/s13287-022-03025-5)
Supplement: Supplementary file 5 — Additional file 5. Primer sequences for qRT-PCR. [file 13287_2022_3025_MOESM5_ESM.doc]

**Table S4. Primer sequences for qRT-PCR.**

| **Gene** | **Forward (5'-3')** | **Reverse (5'-3')** |
| --- | --- | --- |
| eIF5 | TCGATGTTTCGAAGGAGGCT | CTTCTTGTCCCGCTTGTCCT |
| eIF1A | CGAAACCGTCCACCTCTAGT | GGCTTGTTGGCGACCAATTT |
| eIF2γ | AAGCCCAGATCGGTGTGAAC | TTGGCGTTGGCATAACCCAG |
| Ldh | AGAGAAGTGGAACGAGCTGC | ATGCCATGTTCGCCCAAAAC |
| GstE11 | GTTGGACTTGCGACTGGTCA | TCGCCCACCAAGTAATCACC |
| GstD10 | ATTTGATTACCACGTAGTTTCCAG | CGCTGATTGATCAACGCCTG |
| GstD2 | CCGGATCGGATGAGGACTTG | TTCGAACGTGGAGACAGTGG |
| Gapdh | GTGGTGAACGGCCAGAAGAT | GCCTTGTCAATGGTGGTGAA |
