# Supplementary material for: Cyst stem cell lineage eIF5 non-autonomously prevents testicular germ cell tumor formation via eIF1A/eIF2γ-mediated pre-initiation complex
Source: Stem Cell Res Ther. 2022 Jul 26;13:351. doi: 10.1186/s13287-022-03025-5 (PMC9327282; doi:10.1186/s13287-022-03025-5)
Supplement: Supplementary file 6 — Additional file 6. Antibodies used in this study. [file 13287_2022_3025_MOESM6_ESM.doc]

**Table S5. Antibodies used in this study.**

| **Antigen** | **Source** | **Company** | **Application** | **Dilution** |
| --- | --- | --- | --- | --- |
| Vasa | Rat | DSHB | IF | 1:20 |
| 1B1 | Mouse | DSHB | IF | 1:75 |
| Zfh1 | Rat | A gift from Prof. Chao Tong | IF | 1:1000 |
| Eya | Mouse | DSHB | IF | 1:20 |
| PH3 | Rabbit | Cell Signaling Technology | IF | 1:600 |
| FasⅢ | Mouse | DSHB | IF | 1:50 |
| Cyclin A | Mouse | DSHB | IF | 1:50 |
| Cyclin B | Mouse | DSHB | IF | 1:50 |
| HA | Rabbit | Cell Signaling Technology | IF | 1:600 |
| Orb | mouse | DSHB | IF | 1:50 |
